# Reproductive colonization of land by frogs: Embryos and larvae excrete urea to avoid ammonia toxicity

**DOI:** 10.1002/ece3.8570

**Published:** 2022-02-14

**Authors:** Javier Méndez‐Narváez, Karen M. Warkentin

**Affiliations:** ^1^ 1846 Department of Biology Boston University Boston Massachusetts USA; ^2^ Calima Fundación para la Investigación de la Biodiversidad y Conservación en el Trópico Cali Colombia; ^3^ Smithsonian Tropical Research Institute Panama Republic of Panama

**Keywords:** Centrolenidae, dehydration risk, developmental physiology, Leptodactylidae, phenotypic plasticity, Phyllomedusidae

## Abstract

Vertebrate colonization of land has occurred multiple times, including over 50 origins of terrestrial eggs in frogs. Some environmental factors and phenotypic responses that facilitated these transitions are known, but responses to water constraints and risk of ammonia toxicity during early development are poorly understood. We tested if ammonia accumulation and dehydration risk induce a shift from ammonia to urea excretion during early stages of four anurans, from three origins of terrestrial development. We quantified ammonia and urea concentrations during early development on land, under well‐hydrated and dry conditions. Where we found urea excretion, we tested for a plastic increase under dry conditions and with ammonia accumulation in developmental environments. We assessed the potential adaptive role of urea excretion by comparing ammonia tolerance measured in 96h‐LC_50_ tests with ammonia levels in developmental environments. Ammonia accumulated in foam nests and perivitelline fluid, increasing over development and reaching higher concentrations under dry conditions. All four species showed high ammonia tolerance, compared to fishes and aquatic‐breeding frogs. Both nest‐dwelling larvae of *Leptodactylus fragilis* and late embryos of *Hyalinobatrachium fleischmanni* excreted urea, showing a plastic increase under dry conditions. These two species can develop the longest on land and urea excretion appears adaptive, preventing their exposure to potentially lethal levels of ammonia. Neither late embryos of *Agalychnis callidryas* nor nest‐dwelling larvae of *Engystomops pustulosus* experienced toxic ammonia levels under dry conditions, and neither excreted urea. Our results suggest that an early onset of urea excretion, its increase under dry conditions, and elevated ammonia tolerance can all help prevent ammonia toxicity during terrestrial development. High ammonia represents a general risk for development which may be exacerbated as climate change increases dehydration risk for terrestrial‐breeding frogs. It may also be a cue that elicits adaptive physiological responses during early development.

## Graphical abstract

Frogs have evolved terrestrial eggs many times, offering an excellent opportunity to assess traits that may facilitate aquatic‐to‐terrestrial transitions. Our results suggest the importance of physiological adaptations and physiological plasticity during early life stages, specifically the early onset of urea excretion, its upregulation under dry conditions, and elevated ammonia tolerance. All of these can help to prevent ammonia toxicity under water constraints on land, which is both a very general problem facing terrestrial animals that must be solved by new colonists from aquatic lineages, and a challenge exacerbated by climate change.

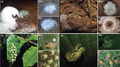

## Resumen

La colonización del medio terrestre ha ocurrido en múltiples ocasiones, incluyendo más de 50 orígenes de oviposición terrestre en ranas. Algunos factores ambientales y respuestas fenotípicas que facilitaron estas transiciones se conocen, pero las respuestas a la restricción al agua y el riesgo de toxicidad por el amonio durante el desarrollo temprano no son bien entendidas. Examinamos si la acumulación de amonio y el riesgo de deshidratación inducen el cambio de excreción de amonio a urea en estadios tempranos del desarrollo de cuatro especies de anuros, en tres orígenes de desarrollo en tierra. Cuantificamos la concentración de amonio y urea durante el desarrollo temprano en tierra en condiciones de hidratación adecuada y de desecación. Cuando encontramos excreción de urea, evaluamos su incremento plástico en condiciones de desecación y de acumulación de amonio en los ambientes de desarrollo. Evaluamos el papel adaptativo de la excreción de urea comparando la tolerancia al amonio medida en pruebas LC_50_ por 96h con los niveles de amonio acumulados en los ambientes de desarrollo. El amonio se acumuló en los nidos de espuma y el fluido perivitelino, incrementando durante el desarrollo y alcanzando la concentración más alta en condiciones de desecación. Las cuatro especies presentan una elevada tolerancia al amonio al compararlas con peces y ranas con reproducción acuática. Las larvas nidícolas de *Leptodactylus fragilis* y los embriones avanzados de *Hyalinobatrachium fleischmanni* excretaron urea, mostrando un incremento plástico en condiciones de desecación. Estas dos especies pueden tener los periodos más extensos de desarrollo en tierra y la excreción de urea parece adaptativa, al prevenir su exposición a niveles de amonio potencialmente letales. Ni los embriones avanzados de *Agalychnis callidryas* ni las larvas nidícolas de *Engystomops pustulosus* experimentan niveles tóxicos de amonio en condiciones de desecación, y tampoco excretaron urea. Nuestros resultados sugieren que un inicio temprano en la excreción de urea, su incremento en condiciones de desecación y una elevada tolerancia al amonio pueden ayudar a prevenir la toxicidad del amonio durante el desarrollo terrestre. El amonio elevado es un riesgo común para el desarrollo que puede ser exacerbado a medida que el cambio climático incrementa el riesgo de desecación en ranas con reproducción terrestre. También puede ser una señal que lleva a respuestas fisiológicas adaptativas durante el desarrollo temprano.

## 1 INTRODUCTION

Vertebrate colonization of land was a major evolutionary event that exposed animals to new ecological and physiological challenges (Bray, 1985). Most aquatic vertebrates excrete nitrogen in the form of cheap, water‐soluble ammonia (Cragg et al., 1961; Wright & Fyhn, 2001). However, living on land increases the risk of dehydration and the need to conserve water creates a waste‐disposal problem (Jørgensen, 1997; Shoemaker et al., 1969), increasing the risk of ammonia toxicity (Chew & Ip, 2014; Ip & Chew, 2010). The shift from excreting ammonia to the less toxic urea is hypothesized to be a key adaptation that facilitated the transition from water to land (Amemiya et al., 2013; Mommsen & Walsh, 1989). One successful approach to understand the environmental challenges and phenotypic responses involved in colonizing land focuses on extant species whose lives cross the water–land interface (Ashley‐Ross et al., 2013; Graham & Lee, 2004; Martin & Carter, 2013; Wright & Turko, 2016). Although urea synthesis occurs in several vertebrate lineages and evolved in multiple environmental contexts (Anderson, 2001; Chew et al., 2004; Costanzo & Lee, 2005; Jørgensen, 1997; Randall et al., 1989; Saha & Ratha, 1989; Shoemaker & Nagy, 1977; Wright et al., 2004), its regulation during early development has not been addressed in the context of the colonization of land.

Developmental plasticity is hypothesized to facilitate colonization events and evolutionary change (Ghalambor et al., 2007; Lande, 2015; West‐Eberhard, 2003) and may have played a role in transitions from aquatic to terrestrial environments. Environmentally induced traits may allow survival in altered developmental environments (Gomez‐Mestre & Buchholz, 2006; Kulkarni et al., 2017; Lande, 2009, 2014), including early colonization of land by tetrapods (Standen et al., 2014). Assessing plasticity in physiological regulatory systems may help to clarify key developmental mechanisms that allow persistence in changing environments (Rundle & Spicer, 2016; Spicer & Burggren, 2003). Few studies have examined environmental regulation of the physiological mechanisms of urea excretion in vertebrates, but some attempts have been made in aquatic organisms (Chadwick & Wright, 1999; Gomez‐Mestre et al., 2004; Janssens, 1972; Wright et al., 1995; Wright & Wright, 1996).

Studies of reproductive biology in amphibians have helped to identify key ecological and environmental conditions that may have allowed evolutionary colonization of land (Haddad & Prado, 2005; Liedtke et al., 2017; Méndez‐Narváez et al., 2015; Touchon & Worley, 2015; Zamudio et al., 2016). In frogs, evolutionary transitions from aquatic to terrestrial breeding have occurred many times, with at least 56 independent origins of terrestrial eggs (Goin, 1960; Gomez‐Mestre et al., 2012). Moving anamniotic eggs to land creates a high risk of desiccation (Eads et al., 2012; Mitchell, 2002; Rudin‐Bitterli et al., 2020). It also creates a potential problem of ammonia disposal; since the ammonia produced as embryos metabolize yolk proteins (Jorgensen et al., 2009) may not easily diffuse into the surrounding environment (Dhiyebi et al., 2013). Plastic extensions of terrestrial development, either *in ovo* after hatching competence or as terrestrial larvae, have also evolved in several lineages, sometimes facilitated by parental care (Delia et al., 2019; Warkentin, 2011), favored by enhanced survival in the water (Warkentin, 1995; Willink et al., 2014), or enforced by the need for flooding to access or create an aquatic habitat (Bradford & Seymour, 1985; Downie, 1984, 1994).

In frogs, embryonic and larval adaptations, including plastic responses during early development, have been described in response to biotic and abiotic environmental challenges on land (Alcocer et al., 1992; Bradford & Seymour, 1985; Delia et al., 2014; Downie, 1984; Seymour & Bradford, 1995; Warkentin, 1995, 2007). Plastic responses to common environmental threats during development may help explain repeated evolution of traits among distantly related taxa, via changes in shared underlying mechanisms (Gomez‐Mestre & Buchholz, 2006; Ledón‐Rettig et al., 2010; Warkentin, 2011). In most frogs, a high capacity for urea synthesis seems to be acquired at metamorphosis, congruent with the developmental and physiological transition from water to land (Cohen, 1970; Munro, 1953). Urea excretion has been reported during terrestrial embryonic and larval development (Alcocer et al., 1992; Grafe et al., 2005; Martin & Cooper, 1972; Schindelmeiser & Greven, 1981; Shoemaker & McClanahan, 1973); however, when urea has been measured in parentally constructed developmental environments (Alcocer et al., 1992; Shoemaker & McClanahan, 1973), studies have not distinguished urea produced by parents versus that produced by the offspring. In addition, few studies have examined how environmental conditions during development, such as dehydration risk and ammonia toxicity, may directly induce or selectively favor urea excretion (Gomez‐Mestre et al., 2004; Wright & Wright, 1996), which could elucidate its potential role in the repeated reproductive colonization of land by amphibians.

Here, we take advantage of the repeated evolution of terrestrial development in frogs to test the hypothesis that facultative urea excretion during early development occurs in response to dry conditions, preventing ammonia accumulation to toxic levels. We further hypothesize that species with longer periods of terrestrial development—whether enforced or opportunistic—are more likely to exhibit urea excretion. To test these hypotheses, we measured the concentrations of ammonia and urea within developmental environments (i.e., eggs and foam nests) across embryonic and early larval stages under ecologically relevant wet (well‐hydrated) and dry conditions for four species. We estimated the amount of ammonia and urea per individual that was present at late terrestrial stages and tested for plasticity in response to dry conditions. Finally, we estimated LC_50_ values for late embryos and early larvae and compared them with ammonia concentrations measured in developmental environments and predicted (potential) to occur in the absence of urea excretion.

### 1.1 Study species

We studied four frog species, from three lineages that independently evolved terrestrial development, where variable extensions of development on land occur and dehydration risk is a common source of mortality (Delia et al., 2013; Méndez‐Narváez et al., 2015; Salica et al., 2017; Zina, 2006). These included two species from the family Leptodactylidae, in which parents create foam nests—a non‐aquatic environment for eggs—with variable degrees of terrestriality (Heyer, 1969; de Sá et al., 2014). In *Engystomops pustulosus*, embryos develop in foam nests floating on ephemeral pools and nest‐dwelling hatchlings can tolerate a short period of extended terrestrial developmental if pools dry and refill (Dalgetty & Kennedy, 2010). In *Leptodactylus fragilis*, eggs and hatchlings develop in nests within subterranean chambers and require rainfall to flood their chamber before moving into an ephemeral pond. These larvae can arrest development and survive for extended periods on land, within larval‐created foam nests (Méndez‐Narváez, 2022). In both of these leptodactylids, extended terrestrial development is enforced by lack of access to an aquatic habitat.

We also studied two species whose embryos develop in arboreal gelatinous clutches that are parentally provisioned with water, with larvae that drop into water upon hatching. In *Agalychnis callidryas* (Phyllomedusidae), females hydrate their clutches at oviposition (Pyburn, 1970). Their embryos can extend terrestrial development *in ovo* by about 80% after reaching hatching competence, if rainfall and humidity maintain adequate egg hydration (Warkentin et al., 2017). In *Hyalinobatrachium* *fleischmanni* (Centrolenidae), males hydrate clutches throughout development (Delia et al., 2013), and hatching‐competent embryos can extend their development *in ovo* by almost 200% under continued care (Delia et al., 2013). In both species, extended terrestrial development is opportunistic; under safe conditions, embryos delay hatching to increase growth and development *in ovo*, which benefits the larvae (Delia et al., 2019; Warkentin, 1995; Willink et al., 2014), while adverse conditions induce earlier hatching (Delia et al., 2014; Salica et al., 2017; Warkentin, 1995).

## 2 METHODS

### 2.1 Study site

We conducted field work in Gamboa Panamá, at and near the Experimental Pond (9°07′14.8″N, 79°42′15.4″W) and along streams crossing Pipeline Road in Soberanía National Park (9°04′33.7″N, 79°39′33.2″W). We collected foam nests and gelatinous clutches from breeding sites during the 2016–2019 rainy seasons with permission from the Panamanian Ministry of the Environment (MiAmbiente permits SC/A‐26‐16, SE/A‐56‐17, SC/A‐51‐18, SE/A‐25‐19). We conducted experiments in an open‐air laboratory, at ambient temperature and humidity (~26.5°C, ~85%), at the Smithsonian Tropical Research Institute (STRI) in Gamboa, with approval from the STRI Animal Care and Use Committee (IACUC protocol # 2016‐0520‐2019A1–A3). We performed laboratory analysis at STRI facilities in Gamboa and Panama City.

### 2.2 Experimental manipulation of hydration

**FIGURE 1:**
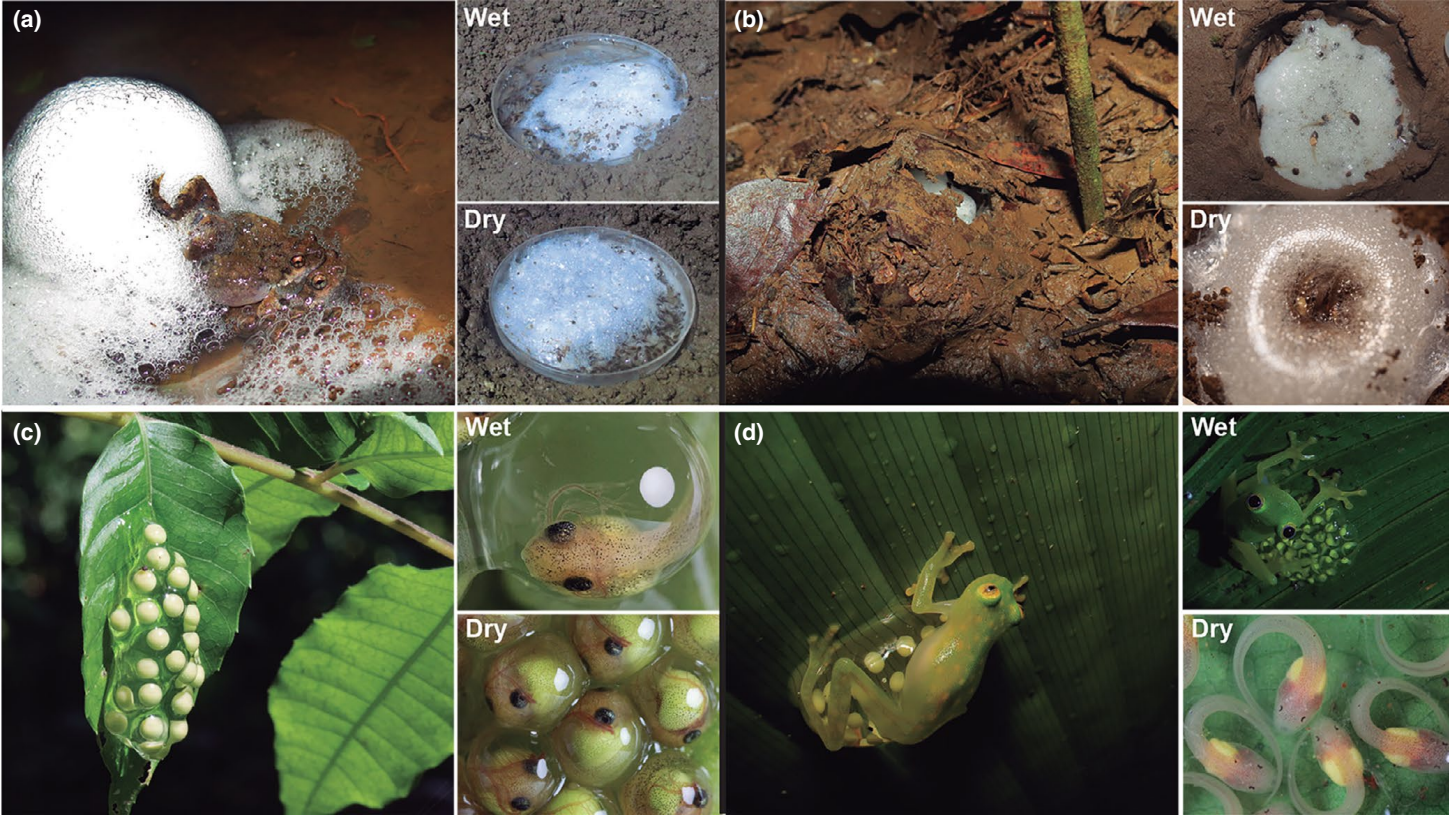
Study species represent four reproductive modes and three origins of terrestrial eggs, and all commonly face the risk of dehydration. We exposed aquatic foam nests of *Engystomops pustulosus* (a), terrestrial foam nests of *Leptodactylus fragilis* (b), terrestrial gelatinous clutches of *Agalychnis callidryas* that lack parental care (c) and terrestrial gelatinous clutches of *Hyalinobatrachium fleischmanni* that require parental care (d) to well‐hydrated (wet) and dry conditions

We exposed egg clutches to ecologically relevant, species‐specific wet and dry conditions in the laboratory, based on their natural history. We collected foam nests of *E*. *pustulosus* from ephemeral pools in the field or by keeping amplectant pairs overnight in small containers with water‐filled Petri dishes. We kept wet nests on water in dishes and, to simulate a dry pool for the dry treatment, we removed the water from dishes with a pipette the morning following oviposition (Figure 1a).

For *L*. *fragilis*, we monitored territories for mating activity and excavated their burrows to collect nests the morning after oviposition. We also measured soil water content near nests using a soil‐moisture smart sensor (S‐SMC‐M005, HOBO^®^). In the laboratory, we buried foam nests in soil collected from breeding sites (Figure 1b). For wet nests, we matched soil water content to that measured near burrows (field conditions, mean ± SD: 0.386 ± 0.05 m^3^/m^3^, *N* = 30), spraying the soil with water daily to maintain hydration (control lab conditions: 0.35–0.40 m^3^/m^3^). For the dry treatment, we reduced soil water content to simulate an extended period without rain (dry lab conditions: 0.25–0.30 m^3^/m^3^).

We collected *A*. *callidryas* egg clutches the morning after oviposition and transported them to the laboratory on the leaves where they were laid. We mounted the clutches (leaves) on plastic cards, set them over water in cups, and housed them in plastic containers with partly screened lids. To simulate ideal field conditions for the wet treatment, we used an automated system to mist clutches hourly with rainwater, removing excess water twice daily to avoid submerging eggs. For the dry treatment, we manually misted clutches daily, providing just enough hydration to avoid mortality while exposing embryos to sublethal dehydration (Salica et al., 2017, Figure 1c).

Because *H*. *fleischmanni* deposit egg clutches on the underside of leaves, embryos are protected from rain and depend on paternal care for hydration (Delia et al., 2013, 2020). Males deliver water via brooding eggs with their pelvic patch; this is required for embryo survival during early development and not effectively replaced by misting. Therefore, we allowed all clutches to receive parental care in the field for 5 days. We then collected clutches for the dry treatment and maintained them in the laboratory for 5 more days, providing minimal manual misting as above. We left wet clutches in the field with their fathers for an additional 5 days of care, then collected them (Figure 1d).

### 2.3 N‐waste concentrations over early development in wet and dry environments

#### 2.3.1 Sampling foam, jelly, and perivitelline fluid

**FIGURE 2:**
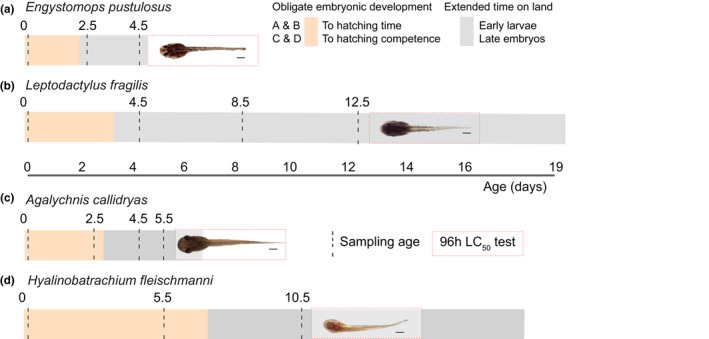
Species‐specific sampling ages (vertical dashed lines) for nitrogen excretion measurements in four frog species, each with a different reproductive mode. After the final N‐waste sample, we conducted 96‐h LC_50_ tests (dashed red box) for ammonia tolerance of tadpoles (image scale = 1 mm) removed from the nest (a, b) or induced to hatch (c, d). All four species have an obligate embryonic period (orange bars) and can extend development on land (gray bars) as nest‐dwelling larvae (a, b) or hatching‐competent embryos (c, d). Larvae must await (re)flooding to leave foam nests (a, b), while embryos (c, d) fall into the water below upon hatching. Because neither embryos nor nest‐dwelling larvae feed, growth through this period occurs only via water absorption and developmental reorganization of maternally provided materials. Growth based on ingestion that increases dry mass begins sometime after tadpoles enter the water

We sampled ammonia and urea concentrations (mmol‐N/L) in developmental environments at multiple times to assess changes over development and the effect of wet versus dry conditions. For a baseline before embryonic excretion, we collected foam (*N* = 12 *E*. *pustulosus* and 11 *L*. *fragilis* nests) or egg jelly (*N* = 9 *A*. *callidryas* and 6 *H*. *fleischmanni* clutches) the morning after oviposition; at this age, these foam nesters are in gastrulation and the arboreal embryos still in cleavage stages. Subsequently, we collected samples of foam and perivitelline fluid (PVF) at species‐specific ages (Figure 2) based on key ecological and behavioral events. Our last sample was timed just before the dry‐treatment constraint of dehydration‐induced mortality in foam nests and hatching in gelatinous clutches.

We used 3‐ml plastic transfer pipettes to collect foam samples from 57 *E*. *pustulosus* and 87 *L*. *fragilis* nests, across multiple ages and experimental conditions. Near‐hatching samples (2.5 days for *E*. *pustulosus*, 4.5 days for *L*. *fragilis*, Figure 2a,b) included embryonic wastes released into the foam at hatching plus some early larval wastes, while samples later during the extended time in the foam included additional larval wastes. We did not directly assess N‐waste loss from nests, nor the stability of wastes within them. We collected foam samples from multiple parts of each large foam nest of *E*. *pustulosus* and most or, at the latest age, all of the foam from the smaller nests of *L*.* fragilis*. Nests of *E*. *pustulosus* and *L*. *fragilis* contained 303 ± 76 (*N* = 11) and 84 ± 24 (*N* = 83) larvae, respectively.

We collected PVF samples from 59 *A*. *callidryas* and 34 *H*. *fleischmanni* clutches with 0.3 ml insulin syringes, pooling PVF extracted from sibling eggs within a clutch. The first PVF samples (2.5 days and 5.5. days for *A*. *callidryas* and *H*. *fleischmanni*, respectively) included early embryonic wastes, before hatching competence. Subsequent samples during the extended time in the egg (Figure 2c,d) also included late embryonic wastes. Clutches of *A*. *callidryas* and *H*. *fleischmanni* contained 34 ± 10 (*N* = 12) and 25 ± 7 (*N* = 15) embryos, respectively.

#### 2.3.2 N‐waste measurements from samples

We froze foam, jelly, and PVF samples at −18°C in microcentrifuge tubes and measured ammonia (total ammonia) and urea concentration within 2 months. We quantified ammonia and urea concentrations using a colorimetric approach (Fawcett & Scott, 1960) with a commercial enzymatic kit (Boehringer Mannheim Cat. No. 10542946035) previously used to quantify these N‐wastes from foam nests (Shoemaker & McClanahan, 1973) and perivitelline fluid (del Pino et al., 1994). We did not assess percent recovery of ammonia and urea from these substances; thus, the values we report, like those reported in earlier work (del Pino et al., 1994; Shoemaker & McClanahan, 1973), could be underestimates. We thawed samples and centrifuged them for 5 min at 16,260 rcf to obtain the liquid portion, measured its volume, and used it for analysis. We assessed ammonia and urea concentrations simultaneously, using 0.2 ml of stock in two quartz cuvettes (0.1 ml each) and measuring changes in absorbance at 340 nm at room temperature with a UV–Visible Spectrophotometer (Thermo‐Scientific Evolution 60S). We ran controls without samples to assess background absorbance of kit reagents for both tests. For double determinations using sample volumes of 0.1 ml, Boehringer Mannheim reports differences of 0.4–1 mg/L for ammonia, and 0.7–2 mg/L for urea (Cat. No. 10542946035). We measured a subset of our samples two or three times to assess measurement precision (ammonia: mean CV = 7.3%, *N* = 21; urea: mean CV = 8.6%, *N* = 9). We report urea‐N and ammonia as molar concentrations (mmol‐N/L), where urea‐N = 2× urea, since each urea molecule has two nitrogen atoms.

For precise measurements with this kit (ΔAbs 0.1–1), test solutions should be in the range of 0.47–4.79 mmol/L (0.008–0.08 g/L) ammonia and 0.23–2.33 mmol/L (0.014–0.14 g/L) urea (Boehringer Mannheim Cat. No. 10542946035). If measured ΔAbs was outside this range and sufficient sample remained, we increased sample volume in test solutions or diluted it with deionized water and repeated the analysis. Because larger samples enable detection of lower concentrations, when sample volume was insufficient to repeat the analysis, we used a less conservative approach, calculating concentrations based on ΔAbs as low as 0.05 (0.23 mmol/L ammonia or urea‐N). When ΔAbs was <0.05 concentration was considered zero. If ΔAbs was too high to measure ammonia concentration, we could not calculate urea concentration either, and the pair of measurements were discarded. If ΔAbs was too high for urea only, we considered it unmeasurable (NA) although urea may have been present; thus, some samples have ammonia measurements without matched urea and total N measurements.

### 2.4 Assessing plasticity in excretion: amounts of N‐wastes at the last sampling age

As an initial approach to test if dry conditions experienced during development may induce a plastic shift from ammonia toward urea excretion, we estimated the amount of ammonia‐N and urea‐N per individual that was present in developmental environments at the latest sampling age for the two species in which early urea excretion was evident. Assessing amount, not concentration, is necessary to distinguish a change in excretion pattern from simple evaporative concentration of wastes. Testing at the last sampling age allows more time for embryos or larvae to express a response to their environment; however, wastes may also be lost from nests or clutches over this time.

For *L*. *fragilis*, we collected all foam and counted larvae in each nest, then centrifuged the foam to separate liquid from air and measured the volume of liquid. There was always less parental foam at 12.5 days than earlier, especially in dry nests; however, in 6 of 18 wet and 5 of 14 dry nests, there was no parental foam visible and larvae had constructed their own nest (Méndez‐Narváez, 2022). These larval nests were too small to pipette foam directly without loss, so we collected the entire nest with a clean spoon, taking care to not collect soil particles, added 0.5 ml of deionized water to dissolve the foam, and collected this fluid for analysis. We could not accurately measure foam fluid volumes for these samples so, conservatively, used the average fluid volume from dry nests (0.02 ml) to estimate the dilution factor to calculate N‐waste concentration. Overestimating volume would yield underestimates of concentration but not change estimates of the amount of N‐wastes present. For *H*. *fleischmanni*, we recorded the number of eggs per clutch that we sampled, extracted all of their PVF, and measured its pooled volume. For each sample, we estimated the total amount of N‐waste (ammonia‐N + urea‐N) per individual accumulated in developmental environments to test if it was affected by dry conditions. We also estimated the potential concentration of ammonia‐N that could have accumulated without urea excretion, assuming that all urea present was produced from ammonia.

### 2.5 Ammonia toxicity in early larval stages

To assess if urea synthesis prevents ammonia toxicity, we conducted standard 96‐h LC_50_ tests for ammonia in all the study species (Figure 2). We selected our initial range of total ammonia nitrogen (TAN) concentrations based on the ammonia levels detected for each species at our latest sampling point in the dry treatment, where we observed no mortality that we could attribute to ammonia toxicity, but ammonia reached the highest levels (Table A1). We ran pilot toxicity trials to adjust concentrations across test solutions for each species in an attempt to cover the full range of mortality (0–100%). For each trial, we randomly assigned 10 tadpoles per concentration into eight experimental TAN concentrations, except in five trials we had fewer concentrations or tadpoles per concentration. We also ran controls in aged (dechlorinated) tap water, for all but three trials, and we never detected mortality. For each TAN concentration, we filled small plastic cups with 15 ml of an experimental solution made from ammonium chloride (NH_4_Cl) in aged tap water.

We tested individuals from our latest sampling age, after extended development on land, after removing them from foam nests or inducing them to hatch from egg clutches. We placed siblings together in aged tap water for 2–6 h to eliminate nitrogen wastes accumulated in the body before the onset of each trial. All *E*. *pustulosus* and all but two *L*. *fragilis* trials used individuals from a single foam nest. For *H*. *fleischmanni* and all but one *A*. *callidryas* trial, we pooled hatchlings from 2 to 3 developmentally matched clutches. To account for intraspecific variation, we ran multiple trials, using tadpoles that came from both wet and dry treatments. In total, we performed 12 trials for *E*. *pustulosus*, 14 for *L*. *fragilis*, 13 for *A*. *callidryas*, and 5 for *H*. *fleischmanni*, some with different TAN concentration ranges (Table A2). Every 24 h, we recorded mortality, removed motionless subjects to a cup with aged tap water to confirm death, and replaced experimental solutions in test cups. Because embryos and foam‐dwelling larvae subsist on yolk, test subjects were unfed. After 96 h, survivors were moved to aged tap water and fed *ad libitum* for at least 1 day before release at their collection site.

### 2.6 Statistical analysis

We conducted statistical analyses using R v 3.6.1 (R Core Team, 2019). Because N‐waste values in several samples fell below detection limits (Table A1) and were considered informative (Douma & Weedon, 2019), we rank‐transformed N‐waste concentrations and amounts to avoid a potential zero‐inflation problem. We accounted for zeros in proportional data with a standard data transformation bounding all values between 0 and 1 (Douma & Weedon, 2019; Smithson & Verkuilen, 2006).

We tested for changes in ammonia and urea concentrations with development (age), treatment (wet and dry conditions), and their interaction, using a permutation approach to obtain *p*‐values (5000 iterations). For *E*. *pustulosus*, we used linear mixed effect models (LMEM), including nest identity (ID) as a random effect to account for some repeated measurements (Bates et al., 2015; *lme4* package) and calculated permutated *p*‐values for fixed factors (Luo et al., 2018; *predictmeans*). For *L*. *fragilis* and *A*. *callidryas*, we tested fixed effects with a permutated ANOVA (Frossard & Renaud, 2019; *permuco*). For *H*. *fleischmanni*, we tested for an effect of age across sampling periods and for treatment only at the last stage. We also performed permutated pairwise comparisons (fdr method), using the corresponding model structure in each case (*predictmeans*). Estimated parametric statistics and *p*‐values are available in Appendix 1 (Tables A3, A4, A5, A6).

We tested for a difference across treatments in the amount of ammonia and urea present in final samples, per individual, using a permutated *t*‐test (Hervé, 2020; *RVAideMemoire*). We also tested if the accumulated ammonia concentration (actual or potential) in developmental environments at the last sampling age contributes or interacts with treatment to predict urea excretion amount, using an AIC approach (Ripley, 2011; *MASS*); if so, we calculated permutated *p*‐values of a LM (*permuco*). Finally, we tested if urea represented a higher proportion of total nitrogen wastes (ammonia‐N+ urea‐N) in dry conditions, using a generalized linear mixed model (GLMMs) with an underlying Beta error distribution (Brooks et al., 2017; *glmmTMB*) and ratio test (LRT) to obtain *p*‐values and confirmation with a permutated *t*‐test (Table A7).

We estimated LC_50_ values for each species by fitting generalized linear models (GLM) using a binomial distribution with a probit‐link function (*lme4*) with TAN concentrations as a fixed effect and used the model to calculate LC_50_ values at 24, 48, 72 h, and 96‐h LC_50_ and their 95% confidence intervals (Hlina, 2020; *ecotox*).

## 3 RESULTS

### 3.1 Ammonia and urea concentrations in terrestrial developmental environments

#### 3.1.1 Foam nesting species

**FIGURE 3:**
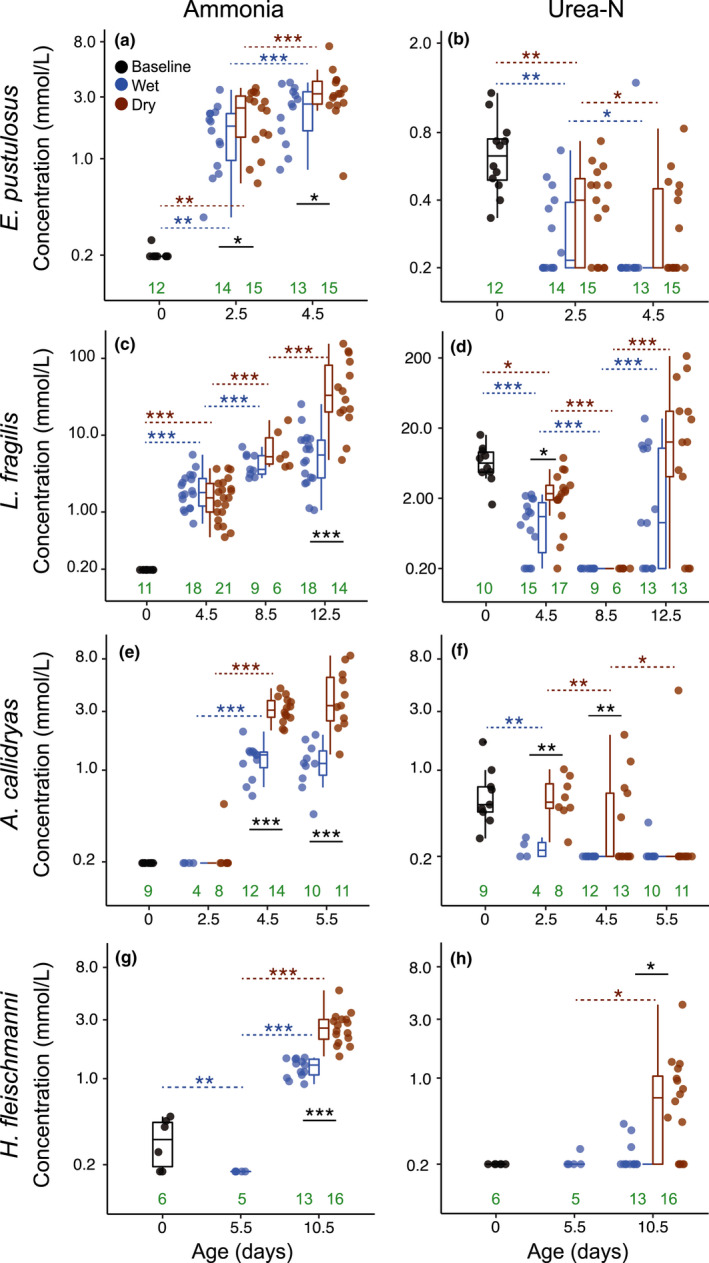
Ammonia and urea‐N concentrations in foam nests, egg jelly, and perivitelline fluid across development for four frog species: (a, b) *Engystomops pustulosus*; (c, d) *Leptodactylus fragilis*; (e, f) *Agalychnis callidryas*; (g, h) *Hyalinobatrachium fleischmanni*. Baseline measurements of N‐wastes (likely of parental origin) were made shortly after oviposition (0 days; black points) from foam and egg jelly. Concentrations over development were measured from foam and PVF under species‐specific wet (blue) and dry (brown) conditions. Box plots show median, first and third quartiles, and extent of data to 1.5 × IQR; data points are also shown. Sample sizes (green) are listed at the bottom of each panel. We obtained *p*‐values by permutation tests for pairwise comparisons (FDR correction), after fitting LMEM (a, b) and LM (c–h) for ammonia and urea concentrations: *p < .05, **p < .01, ***p < .001 (Tables A3–A6). Significant differences between treatments are indicated with solid black lines and changes across consecutive ages with dotted blue (wet) or brown (dry) lines. Note the log scale of *Y*‐axes; for values below the detection threshold of 0.23 mmol/L for ammonia and urea‐N, we assigned the arbitrary sub‐threshold value of 0.2 mmol/L. See Table A1 for descriptive analysis including zeros and total‐N

Ammonia was below the detection limit in newly laid *E*. *pustulosus* nests, with one exception, and all foam nests later accumulated ammonia (Figure 3a, Table A1). Ammonia concentration changed with age (*p* = .0002), and treatment (*p* = .01), with no interaction effect (*p* = .76). Its concentration increased in foam nests from oviposition to 2.5 days (near hatching) and, again to 4.5 days (extended development) in both wet and dry treatments (Figure 3a, Table A3). Ammonia concentration was higher in dry foam nests at both ages (Figure 3a, Table A3). We found urea in all newly laid foam nests. Urea concentration changed with age (*p* = .0002) and treatment (*p* = .03), with no interaction effect (*p* = .59). Its concentration decreased after oviposition with more nests falling below the urea detection limit over time, but with a marginally higher concentration in dry conditions (Figure 3b, Table A3).

Ammonia was undetectable in newly laid *L*. *fragilis* nests and present in all older nests (Figure 3c, Table A1). Ammonia concentration changed with age (*p* = .0002), treatment (*p* = .02), and their interaction (*p* = .002, Table A4). Its concentration increased in both treatments from oviposition to 4.5 days (near hatching) and again to 8.5 days (first extended development sample). However, it only continued to increase from 8.5 to 12.5 days (second extended development sample) in dry nests. Ammonia concentration was higher in dry conditions at the latest age (12.5 days, Figure 3c, Table A4). We found urea in all recently laid foam nests (Figure 3d, Table A1). Urea concentration changed with age (*p* = .0002), but not with treatment (*p* = .07) or their interaction (*p* = .28). First, its concentration decreased, falling below the detection limit by the first extended development sample (8.5 days). Then, its concentration increased, especially in dry nests, by the second extended development sample (Figure 3d, Table A4). Urea concentration was significantly higher in dry conditions only near hatching (4.5 days, Figure 3d, Table A4).

#### 3.1.2 Arboreal gelatinous clutches

Ammonia was undetectable in the jelly of newly laid *A*. *callidryas* clutches and the PVF at 2.5 days, except for one dry clutch (Figure 3e, Table A1). Ammonia concentration in the PVF changed with age, treatment, and their interaction (all *p* = .0002, Table A5). Its concentration increased more rapidly in dry clutches, from 2.5 days (early development) to 4.5 days (hatching competence), but we detected no change from 4.5 to 5.5 days. However, ammonia concentrations remained higher in dry clutches (Figure 3e, Table A5). We found urea in the jelly of all newly laid clutches (Figure 3f, Table A1). Its concentration changed in the PVF with age (*p* = .0002), treatment (*p* = .001), and their interaction (*p* = .02). Urea concentration decreased over development and fell below detection limits by 4.5 days in wet clutches, when it still remained detectable in the dry treatment. In both treatments, it was undetectable by 5.5 days (extended terrestrial development), with one exception in each condition (Figure 3f, Table A5). Urea concentration was higher in dry conditions at 2.5 and 4.5 days (Figure 3f, Table A5).

Ammonia was detected in the jelly of some, but not all, recently laid *H*. *fleischmanni* clutches (Figure 3g, Table A1). Ammonia concentration in the PVF differed among age and treatment categories (*p* = 0.0002). It was undetectable after 5.5 days of paternal care in the field (1.5 days before hatching competence). Ammonia concentration then increased as development continued in both treatments, but concentrations were greater in the dry treatment without fathers (Figure 3g, Table A6). Urea was undetectable in the jelly of newly laid clutches (Figure 3h, Table A1). Urea concentration in the PVF differed among age and treatment categories (*p* = .002). At 5.5 days (before hatching competence), urea was detectable in the PVF of only one clutch. However, by 10.5 days (3.5 days into extended terrestrial development) urea was detected in more clutches and significantly higher in the dry treatment (Figure 3h, Table A6).

### 3.2 Amount of urea in relation to dehydration and potential ammonia concentration

**FIGURE 4:**
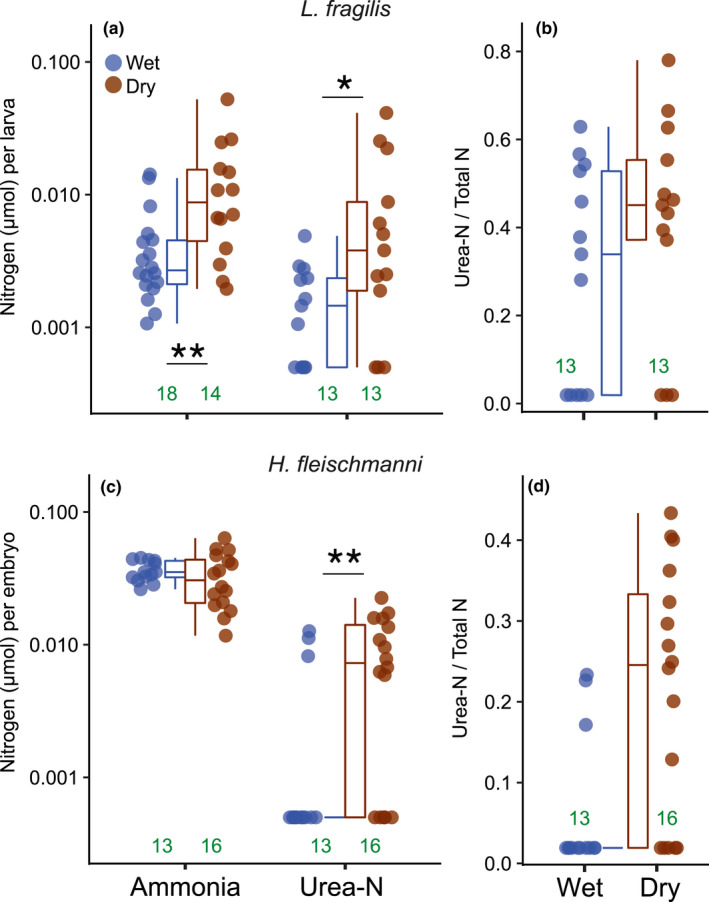
Amounts of ammonia‐N and urea‐N present in developmental environments per individual for (a) early larvae of *Leptodactylus fragilis* and (c) late embryos of *Hyalinobatrachium fleischmanni*, and the proportion of total nitrogen waste (ammonia +urea‐N) present as urea for both species (b, d) after development in wet (blue) and dry (brown) conditions to the latest sampling age (12.5 days and 10.5 days, respectively). *p*‐Values were obtained from permutation tests *p < .05, **p < .01, ***p < .001 (Table A7). Sample sizes are included at the bottom of each panel, in green. The lowest plotted urea‐N amounts (a, c) are arbitrary sub‐threshold values indicating urea was undetectable

We found evidence of embryonic/larval urea excretion in *L*. *fragilis* and *H*. *fleischmanni* that we could separate from baseline urea (Figure 3). We also found that dry foam nests and jelly clutches contained less fluid than those in the wet treatment (*L*. *fragilis* nests: 0.02 ± 0.01 ml, *N* = 8 vs. 0.11 ± 0.06 ml, *N* = 13; *t*_16.4_ = 3.91, *p* = .001; *H*. *fleischmanni* clutches: 0.29 ± 0.1 ml, *N* = 14 vs. 0.61 ± 0.2 ml, *N* = 6; *t*_6.18_ = 3.32, *p* = .0152). Fluid volume was negatively correlated with ammonia and urea concentrations in foam nests of *L*. *fragilis* (ammonia: S = 2638, *p* = .0003; urea: S = 1757, *p* = .0167) and with ammonia but not urea concentration in the PVF of *H*. *fleischmanni* (ammonia: S = 2175.5, *p* = .0026; urea: S = 1633.7, *p* = .3329). Therefore, we calculated the amount of ammonia and urea present per individual in our latest samples of these two species, to assess the possibility of plastic changes (Figure 4).

**FIGURE 5:**
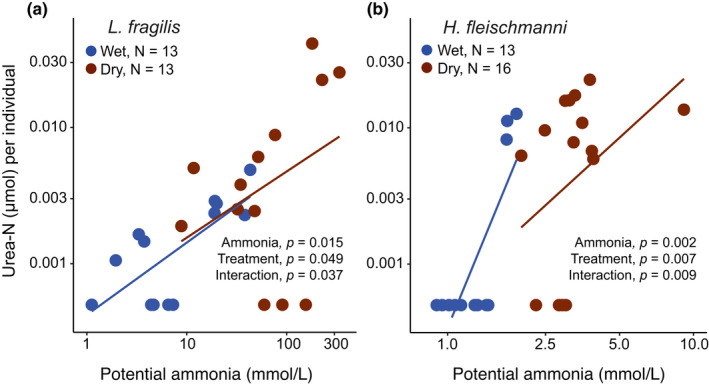
Amount of urea‐N present per individual *Leptodactylus fragilis* larva (a) and *Hyalinobatrachium fleischmanni* embryo (b) in relation to the potential ammonia concentration that could be present in their developmental environments without the urea cycle, under wet (blue) and dry (brown) conditions, at the latest sampling age (12.5 days and 10.5 days, respectively). Calculations of potential ammonia assume that all urea was produced from ammonia. *p*‐Values were obtained from permutation tests from a LM in both species (Table A8)

In *L*. *fragilis*, the amount of ammonia present per individual differed between treatments (*p* = .008; Table A7, Figure 4a), and urea was higher in the dry treatment (*p* = .049, Table A7, Figure 4a). However, the proportion of total nitrogen waste present as urea did not differ between wet and dry foam nests (Table A7, Figure 4b). In analyses including actual ammonia concentration, there was no ammonia or interaction effect on urea per individual (Table A8). However, higher potential ammonia concentration (*p* = .015; Table A8, Figure 5a) and dry conditions (*p *= .049) both increased the per‐individual amount of urea present, with a significant interaction (*p* = .037). Furthermore, we found that dry foam nests had a higher total amount of N‐waste present per individual than did wet nests (dry: 23.01 ± 18.52 μmol, *N* = 13 vs. wet: 6.45 ± 3.76 μmol, *N* = 13; *p* = .005, Table A7).

In *H*. *fleischmanni*, the per‐individual ammonia present did not differ between treatments (Table A7, Figure 4c), but urea was higher in the dry treatment (*p* = .022). Moreover, the proportion of total nitrogen wastes as urea was higher in dry clutches (*p* = .006, Table A7, Figure 4d). Higher potential ammonia concentration and dry conditions both increased urea amounts, with a significant interaction (ammonia *p* = .002, treatment *p* = .007, interaction, *p* = .009; Table A8, Figure 5b). Actual ammonia concentration did not explain urea amounts in this species, neither as a main effect nor an interaction with treatment (Table A8). Furthermore, total N‐wastes per individual did not differ between wet and dry conditions (dry: 41.49 ± 16.11 μmol, *N* = 13 vs. wet: 38.67 ± 9.99 μmol, *N* = 16, *t* = −1.21 *p* = .669, Table A7).

### 3.3 Ammonia tolerance (LC_50_) in early life stages during terrestrial development

**FIGURE 6:**
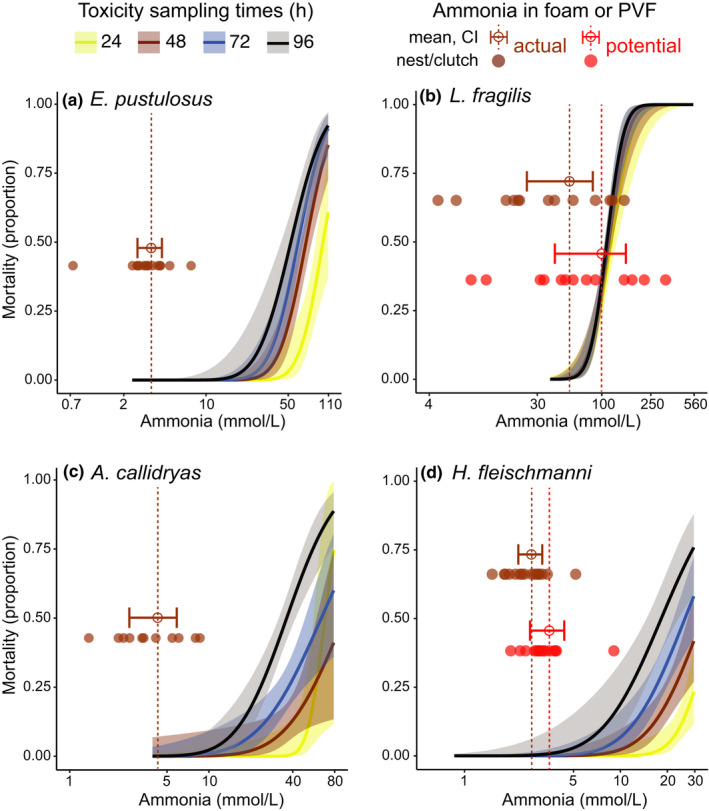
Relationship between ammonia tolerance and the actual and potential ammonia levels in developmental environments for four frog species (a–d). LC_50_ curves represent mortality as a function of ammonia (TAN) concentration for four exposure durations (color coded). Curves (solid) for each exposure duration were fit using adjusted binomial functions (probit link); their 95% confidence intervals are shaded. Ammonia concentrations in foam nests and perivitelline fluid at the last sampling age, under dry conditions, are plotted in brown. Data points represent individual nests or clutches; means are shown with open circles and vertical dashed lines, error bars are 95% CI. For species where urea was present (b, d) the corresponding data for potential ammonia concentration, if all urea‐N were present as ammonia, are shown in red. Note that the *X*‐axis range differs among species

Early larvae of *L*. *fragilis* had the highest LC_50_ level at 24 and 96 h (120 and 110 mmol/L, respectively; Table 1), while *H*. *fleischmanni* had the lowest LC_50_ value at 24 and 96 h (39 and 18 mmol/L, respectively; Table 1). In general, LC_50_ decreased over time from 24 to 96 h in all study species (Table 1, Figure 6).

**TABLE 1:** Ammonia LC_50_ values (mmol/L) for early larvae of *Engystomops pustulosus*, *Leptodactylus fragilis*, *Agalychnis callidryas*, and *Hyalinobatrachium fleischmanni*

Exposure	24 h	48 h	72 h	96 h
*E*. *pustulosus*: N wet = 9, dry = 3				
LC_50_	99 (86–141)	71 (63–79)	60 (53–68)	53 (43–60)
	*χ* ^2^ = 249.61 *p* < .0001	*χ* ^2^ = 341.71 *p* < .0001	*χ* ^2 ^= 351.99 *p* < .0001	*χ* ^2^ = 304.46 *p* < .0001
*L*. *fragilis*: N wet = 11, dry = 3				
LC_50_	120 (113–132)	114 (108–122)	110 (105–123)	110 (105–116)
	*χ* ^2^ = 233.91 *p* < .0001	*χ* ^2^ = 263.74 *p* < .0001	*χ* ^2^ = 306.2 *p* < .0001	*χ* ^2^ = 314.01 *p* < 0.0001
*A*. *callidryas*: N wet = 8, dry = 5				
LC_50_	62 (57–393)	94 (59–58318)	63 (48–142)	36 (31–42)
	*χ* ^2^ = 144.88 *p* < .0001	*χ* ^2^ = 85.99, *p* < .0001	*χ* ^2^ = 129.4, *p* < .0001	*χ* ^2^ = 246.01 *p* < .0001
*H*. *fleischmanni*: N lab = 5, field = 2 (all wet)				
LC_50_	39 (31–136)	33 (27–62)	26 (22–36)	18 (15–24)
	*χ* ^2^ = 42.02, *p* < .0001	*χ* ^2^ = 74.64, *p* < .0001	*χ* ^2^ = 108.29 *p* < .0001	*χ* ^2^ = 152.22, *p* < .0001

Note

Ammonia LC_50_ was estimated at 24‐h intervals until 96 h after water entry. LC_50_ values and their 95% confidence intervals (CI) were estimated for the best model using the *ecotox* package; model fit is below. Trials included individuals from both wet and dry nest/clutch treatments, except for *H. fleischmanni* where all clutches had experienced high hydration, in the field and laboratory; sample sizes of trials from each rearing treatment are indicated.

In all four species, even under dry conditions, the mean ammonia concentration in developmental environments (foam nests or PVF; Figure 3, Table A1) at the latest sampling age was below the 95% CI of LC_50_ values (Table 1) and below the concentration range that caused mortality (Figure 6). However, several dry *L*. *fragilis* nests and one dry *H*. *fleischmanni* clutch had ammonia concentrations in the range that could cause mortality (Figure 6b,d), and levels in a few dry *A*. *callidryas* clutches reached the onset of mortality (Figure 6c). For the two species where urea excretion was evident, we calculated the potential ammonia concentrations that could occur if all urea‐N present were in the form of ammonia. In dry *L*. *fragilis* nests, the mean potential ammonia level was slightly below the LC_50_, with the 95% CI extending to near 100% mortality (Figure 6b). In dry *H*. *fleischmanni* clutches, the 95% CI for potential ammonia overlapped with the onset of mortality at 96 h (Figure 6d).

## 4 DISCUSSION

In frogs from two lineages that independently colonized land for reproduction, urea accumulates around early stages during terrestrial development, revealing an embryonic/larval capacity for urea excretion. Ammonia also accumulates over development in terrestrial environments, reaching a higher concentration under dry conditions in all four species tested (Figure 3, Table A1). Urea excretion was evident in species whose early stages spend longer on land, in nest‐dwelling early larvae of *L*. *fragilis* and late embryos of *H*. *fleischmanni*. In both, we found greater amounts of urea per individual in drier developmental environments, in foam nests in dry versus wet soil (Figure 4a) and egg clutches without versus with parental care (Figure 4b), suggesting N‐excretion plasticity. This may be mediated by waste concentration, since the amount of urea increases as total N‐waste, and thus potential ammonia concentration rises (Figure 5). Urea excretion appears to prevent the accumulation of toxic ammonia concentrations during extended development on land, especially under dry conditions where potential ammonia levels are more likely to enter the lethal range (Table 1; Figure 6b,d). For all species, measured ammonia concentrations in developmental environments (Table A1) remained below the lethal range (Table 1, Figure 6); this could explain the apparent lack of urea excretion by *E*. *pustulosus* and *A*. *callidryas*. The variation in urea accumulation among and within species, and likely congruent variation in urea excretion, suggests that it is not simply terrestrial development but more specifically the risk of ammonia toxicity on land (Figure 6) that regulates urea synthesis. Our results also suggest that plasticity in urea excretion may facilitate extended terrestrial development in some contexts, by preventing toxic levels of ammonia under the common risk of dehydration.

### 4.1 Ammonia accumulation in developmental environments

**FIGURE 7:**
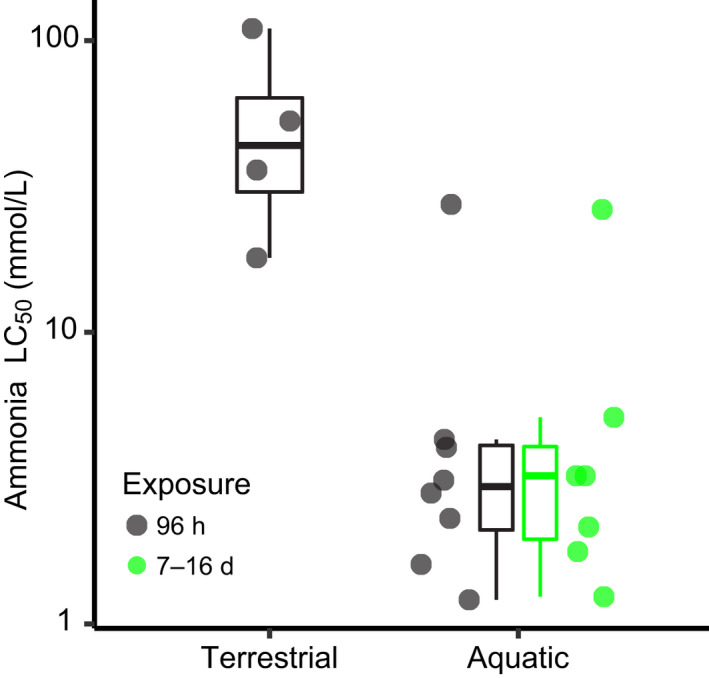
96‐h LC_50_ values for early life stages of foam‐nesting and arboreal‐breeding frogs, from this study (terrestrial) compared to 96‐h and chronic LC_50_ values reported for embryos and larvae of aquatic‐breeding frogs (aquatic). Only *Bufo bufo* (Xu & Oldham, 1997) is more ammonia tolerant than *Hyalinobatrachium fleischmanni*, the most sensitive of the four we studied. See Table A9 for references and species

As expected, our data support that terrestrial development, particularly when prolonged, can generate a waste‐disposal problem, as ammonia accumulates in developmental environments (Figure 3). This ammonia is presumably the product of protein breakdown from yolk reserves used in embryonic and early larval differentiation, as reported in fishes (Dworkin & Dworkin‐Rastl, 1991; Finn et al., 1995) and *Xenopus* (Jorgensen et al., 2009). We detected ammonia levels up to 8.51, 7.40, and 5.20 mmol/L in *A*. *callidryas*, *E*. *pustulosus*, and *H*. *fleischmanni*, respectively (Figure 3), and above 155 mmol/L in *L*. *fragilis*, with higher concentrations under dry conditions. These values of ammonia exceed both assessed tolerances (i.e., LC_50_) reported for tadpoles of aquatic‐breeding species (Figure 7, Table A9) and levels considered safe to avoid toxicity in freshwater environments (acute, 1.0 mmol/L TAN; chronic, 0.11 mmol/L TAN; EPA, 2013). Our results reveal that, even in a rainforest context, terrestrial development exposes early life stages of anurans to ammonia levels that can produce toxic effects in many species. The drier conditions that occur during short periods without rainfall during the reproductive (rainy) season, which are increasingly frequent with climate change, may exacerbate this (Lowe et al., 2021; Touchon & Warkentin, 2009).

### 4.2 Early onset of urea excretion during terrestrial development – context matters

The higher risk of ammonia toxicity is considered a key factor that selects for urea excretion when vertebrates move onto land (Wright, 1995; Wright & Turko, 2016), and transitions to terrestrial development are widespread in frogs, particularly in wet tropical environments (Gomez‐Mestre et al., 2012). Amphibians that move to land at metamorphosis are able to shift from ammonia to urea excretion at that stage (Cohen, 1970; Munro, 1953). Finding urea in perivitelline fluid and foam nests, as we did in all four of our study species (Figure 3), suggests its excretion by early life stages but, alone, is not definitive evidence. Although urea was undetectable in newly laid *H*. *fleischmanni* clutches, it was present in *A*. *callidryas* clutches and foam nests of both *Leptodactylus* species long before the onset of embryonic ammonia excretion. The urea present in newly laid clutches and nests was presumably deposited by the ureotelic adult frogs, perhaps with fluid from the female's bladder used to hydrate clutches at oviposition (Pyburn, 1970). This urea gradually disappeared from nests and clutches as embryos developed (Figure 3); it may have entered the surrounding environment with water movement, as suggested for *L*. *bufonius* (Shoemaker & McClanahan, 1973). Importantly, measurements at a single later stage cannot distinguish parental versus embryonic sources of urea. This is relevant for at least some studies that reported urea in PVF and foam nests during terrestrial development (Alcocer et al., 1992; del Pino et al., 1994; Shoemaker & McClanahan, 1973). Other studies reported an early capacity for urea excretion based on indirect measurements, after initially terrestrial individuals were moved to water (Grafe et al., 2005; Martin & Cooper, 1972; Shoemaker & McClanahan, 1982), or arginase activity was detected in embryonic tissues (Alcocer et al., 1992; Shoemaker & McClanahan, 1982). Nonetheless, arginase can be involved in other biochemical pathways during early development, so activity of rate‐limiting enzymes such as CPS1 is necessary to confirm urea cycle activity (Srivastava & Ratha, 2010). Our finding that urea is present in terrestrial nests and clutches of some anurans without, or before, embryonic or larval excretion suggests that, beyond detection, measures of changes in urea concentration over development will be necessary to clarify the distribution and prevalence of the capacity for urea excretion at early life stages, as well as its potential environmental regulation.

Our results support urea synthesis by embryos of *H*. *fleischmanni*, where urea was absent in early development and higher amounts accumulated in clutches removed from parental care. We also found urea synthesis by early larvae of *L*. *fragilis* during extended development in foam nests (8–12 days), after parental urea disappeared. At this stage, these larvae, like those of *L*. *fuscus*, can produce their own foam if needed (Downie, 1984; Méndez‐Narváez, 2022); our measurements of urea in new larval foam, produced in a urea‐free environment, provide further evidence for larval urea excretion (Méndez‐Narváez, 2022). Embryos of *H*. *fleischmanni* can remain *in ovo* up to 19 days with reliable paternal care (Delia et al., 2019), while early larvae of *L*. *fragilis* can slow development after 8 days and survive for an extended period on land (2 or 3 weeks; Méndez‐Narváez, 2022). In both cases, the ability to excrete urea may reduce the risk of ammonia accumulation to toxic levels during this extended terrestrial period. We found no evidence for urea synthesis in *A*. *callidryas* or *E*. *pustulosus*, which have shorter total and facultative periods of terrestrial development (Figure 2). Thus, urea excretion may occur and improve survival in certain terrestrial development contexts and be disfavored in others where its costs exceed any benefits (Shambaugh, 1977; Wright & Wright, 1996).

Embryos of *A*. *callidryas* hatch early in response to drying (Salica et al., 2017). The specific cue mediating this response remains unknown, but our results suggest that ammonia is a candidate worth testing. Early hatching has been suggested to occur in fish embryos in response to high ammonia accumulation in the PVF (Wright & Fyhn, 2001). Experiments, to date, do not support this (Steele et al., 2001), but combinations of cues such as ammonia and hypoxia should also be considered (Dhiyebi et al., 2013; Ortiz‐Santaliestra et al., 2010). We obtained samples of *A*. *callidryas* PVF from moderately dry eggs, but embryos can experience—and survive—more extreme egg drying (authors, personal observation). Moreover, ammonia levels in drying *A*. *callidryas* clutches approached lethal levels more closely than they did for *E*. *pustulosus* foam nests. Thus, we cannot reject the possibility that urea synthesis may occur under more extreme dehydration; in other work, we found urea in embryonic tissues of *A*. *callidryas* (Méndez‐Narváez, 2022). These embryos could also employ alternative physiological mechanisms to limit toxic ammonia accumulation in the PVF. For instance, some fish embryos under high environmental ammonia sequester ammonia in the yolk (Braun et al., 2009; Steele et al., 2001) and others remove it by synthesizing glutamine (Essex‐Fraser et al., 2005; He et al., 2010; Sanderson et al., 2010; Wright et al., 2007). Some adults may even avoid ammonia toxicity by using partial amino acid catabolism to produce alanine for an energy source during periods of terrestrial exposure (Ip & Chew, 2010). Nonetheless, the more limited capacity of *A*. *callidryas* to extend development *in ovo*, compared to *H*. *fleischmanni* and other glass frogs (Delia et al., 2019), may limit the benefits these embryos could gain by urea excretion and other ammonia detoxification strategies.

### 4.3 Urea excretion plasticity in response to dry conditions and risk of ammonia toxicity

At late terrestrial stages of both *L*. *fragilis* and *H*. *fleischmanni*, we found higher amounts of urea per individual under dry conditions (Figure 4). Since both foam nests of *Leptodactylus* and gelatinous clutches of *A*. *callidryas* lose the presumably parental urea that was present at early stages (Figure 3), it is unlikely that our late samples capture all of the urea produced by developing young, and we cannot rule out a role for differential loss rates in contributing to final amounts. Nonetheless, our results suggest that these embryos and nest‐dwelling larvae increase urea excretion in response to dry conditions.

Embryos of *H*. *fleischmanni* can remain *in ovo* up to 19 days with care (Delia et al., 2013) or hatch as early as 7 days after parental desertion (Delia et al., 2014). Our results show that parental egg brooding, which provides hydration, prevents the accumulation of high ammonia concentrations within eggs. They also suggest that embryos’ ability to shift from ammonia to urea excretion may help them to cope with the dehydration that can occur without brooding, facilitating a several‐day plastic delay in hatching even without care (Delia et al., 2013, 2020). Embryos of *L*. *fragilis* may reach water and survive as early as 3 days if flooded or hatch at 3.5 days and remain in the foam for weeks. While late embryos of *H*. *fleischmanni* can hatch to escape a deteriorating clutch environment, falling into the stream below, larvae of *L*. *fragilis* cannot leave their burrow until it floods; instead, they arrest development and produce new foam to prolong their survival on land (Downie, 1984; Méndez‐Narváez, 2022). The inability of *L*. *fragilis* larvae to control when they leave the nest, thus higher potential N‐waste accumulation (Figure 3c,g), may explain their greater ureotelism even in wet conditions (on average, almost 40% N‐waste excreted as urea; Figure 4a,b). In contrast, *H*. *fleischmanni* show a strong increase in urea production under dry conditions (Figure 4c,d). Urea was detectable in most of the wet *L*. *fragilis* nests (61% vs. 23% of wet *H*. *fleischmanni* clutches; Figure 4). A subset of these nests, like some dry treatment nests, appeared to have completely lost their parental foam by the last sampling age (JMN, personal observation) and had amounts of urea and potential ammonia concentrations resembling the dry ones (Figure 5a); even so, differences between urea in wet and dry nests were still evident (Figure 4).

Our results support a role for ammonia accumulation, and its potential toxicity, in mediating a plastic increase in urea excretion under dry conditions. In both *L*. *fragilis* and *H*. *fleischmanni*, the ammonia concentration that could have occurred if all N‐waste were present in that form predicted the amount of urea we found (Figure 5; Table A8). Because N‐wastes concentrate as water is lost, if all waste remains as ammonia drying increases risk of toxicity. Experimentally increasing environmental ammonia increases urea synthesis in ureotelic fish species, via upregulation of the activity of urea cycle enzymes (Barimo et al., 2004; Barimo & Walsh, 2005; Chew et al., 2005; Ip, Leong, et al., 2005; Ip, Peh, et al., 2005). In aquatic bullfrog tadpoles, urea excretion increases with environmental ammonia, apparently without upregulating urea cycle enzymes (Wright & Wright, 1996). However, precocious activation of urea cycle enzymes has been reported in embryos of some fishes (Chadwick & Wright, 1999; Kharbuli et al., 2006; Wright et al., 1995), where it is hypothesized to have evolved to prevent high perivitelline ammonia levels where the chorion and water chemistry limit ammonia diffusion (Dhiyebi et al., 2013; Rahaman‐Noronha et al., 1996). Although urea can be produced by other pathways, such as purine degradation (Balinsky, 1972) and arginolysis (Srivastava & Ratha, 2010), we have found that CPSase 1, a rate‐limiting enzyme in the urea cycle, is active in tissues of terrestrial *L*. *fragilis* larvae and *H*. *fleischmanni* embryos (Méndez‐Narváez, 2022). In *L*. *fragilis*, exposure of early larvae to sublethal ammonia levels in water increases urea accumulation in tissues and activity of two urea cycle enzymes, compared to siblings in water (Méndez‐Narváez, 2022). This is consistent with an ammonia‐induced plastic increase in urea excretion as risk of ammonia toxicity increases in terrestrial developmental environments.

We also detected a higher total amount of N‐waste in dry versus wet nests of *L*. *fragilis*, but not in dry versus wet clutches of *H*. *fleischmanni*. Development rates are the same in wet and dry nests of *L*. *fragilis* (Méndez‐Narváez, 2022). However, adaptations that improve survival on land, enabling extended terrestrial development, may involve specific metabolic demands that vary with hydration. We hypothesize that N‐waste production may increase as larval foam production increases in the dry treatment. Larval foam making is a key behavior facilitating extended survival on land (Downie, 1984; Kokubum & Giaretta, 2005) and may require considerable energy for bubble blowing, as well as glycoprotein for mucus production.

Moreover, interspecific variation in ammonia tolerance, combined with risk of ammonia accumulation to toxic levels in terrestrial developmental environments, may explain variation in urea synthesis. For *A*. *callidryas* and *E*. *pustulosus*, we found no evidence for urea excretion, and ammonia levels in their PVF and foam nests did not enter the lethal range (Figure 6a,c). We cannot, however, rule out urea synthesis under more extreme dehydration. These embryos and larvae had a substantial margin of safety under wet conditions and for *E*. *pustulosus* even in dry conditions, but some *A*. *callidryas* clutches were close to the onset of mortality in our dry treatment. In contrast, for both *L*. *fragilis* and *H*. *fleischmanni*, the potential ammonia concentration, if all N‐waste was ammonia, entered the lethal range (Figure 6b,d), reaching as high as 100% predicted mortality in *L*. *fragilis*. Although *L*. *fragilis* larvae can tolerate high ammonia levels, without urea excretion during extended development on land they could face lethal toxicity; thus, we consider their high prevalence of urea excretion and its increase in dry conditions to be adaptive. Embryos of *H*. *fleischmanni*, which require parental care, show much lower ammonia tolerance. Without care, potential ammonia levels can reach the onset of mortality in their toxicity curve (Figure 6d) and, in many clutches, embryos excrete urea. If parental care is intermittent, urea excretion may enable embryos to survive a period of neglect and then benefit from further care, rather than simply hatching to escape a deteriorating egg environment; this plastic increase in urea excretion also appears to be adaptive. Overall, these results support the hypothesis that a plastic increase in urea excretion is associated with the risk of ammonia toxicity under water constraints on land, likely improving survival and facilitating extended development of embryos and early larvae on land.

### 4.4 High ammonia tolerance also prevents ammonia toxicity with development on land

Our results suggest that terrestrial frog embryos/early larvae have evolved substantially higher ammonia tolerance than early life stages of aquatic‐breeding frogs and fishes (Figure 7, Table A9). Like ammonia levels in developmental environments, ammonia tolerance varied among our study species (Table 1), but even the most sensitive, *H*. *fleischmanni*, showed greater tolerance than reported for most other anuran larvae, with the sole exception of *Bufo bufo* (Figure 7, Table A9; Xu & Oldham, 1997). Comparable ammonia tolerances at early stages are also reported for ureotelic embryos of some fishes, in particular the toad fish *Opsanus beta* (Barimo & Walsh, 2005) and *Oncorhynchus mykiss* (Rice & Stokes, 1975). Some adult fishes that live with high environmental ammonia or low water availability also show high ammonia tolerance and synthesize urea, including the African lungfish *Protopterus dolloi* that can survive for prolonged time on land during estivation (Chew et al., 2004. Table A9), the facultative air‐breathing teleost *Heteropneustes fossilis* (Saha & Ratha, 1994; Table A9) and the swamp eel *Monopterus albus* (Ip, Leong, et al., 2005; Ip, Peh, et al., 2005; Table A9). Urea excretion was suggested to prevent toxicity during terrestrial development in two frogs, *Leptodactylus bufonius* and *Gastrotheca riobambae* (del Pino et al., 1994; Shoemaker & McClanahan, 1973). However, toxicity tests found low ammonia tolerance in these tadpoles, with lethal ammonia levels orders of magnitude lower than the levels reported in their developmental environments (Table A9). This mismatch and potential methodological limitations of the studies (e.g., chronic ammonia exposure and high pH) suggest a need to re‐examine these species (Brinkman et al., 2009; Thurston et al., 1981) and limit comparisons with other research. A pattern similar to our results occurs in *O*. *beta* and *Oncorhynchus mykiss*, where ammonia levels in their developmental environments (nests and PVF; Table A9) do not reach toxic levels, most likely due to their early urea excretion (Barimo & Walsh, 2005; Dhiyebi et al., 2013; Rice & Stokes, 1975).

### 4.5 Evolutionary implications of early onset of urea excretion in vertebrates

The transition from aquatic to terrestrial life at metamorphosis in anurans (Wilbur & Collins, 1973), and concurrent shift from ammonotelism to ureotelism (Munro, 1953), has been a strong focus of ecological, physiological, and evolutionary research (Laudet, 2011; Lowe et al., 2021; Wassersug, 1975). However, frogs have evolved a wide array of life‐history traits and parental strategies that allow them to reproduce and develop out of water (Gomez‐Mestre et al., 2012; Haddad & Prado, 2005). Our results suggest that reproductive colonization of land by frogs was enabled not only by parental adaptations, such as water provisioning and thermal buffering of eggs (Delia et al., 2020; Méndez‐Narváez et al., 2015; Pyburn, 1970), but also by embryonic and larval physiological adaptations. These include physiological responses to terrestrial conditions, such as an early onset of urea excretion, its upregulation under dry conditions, and elevated ammonia tolerance, all of which can help to prevent ammonia toxicity under water constraints. Such physiological traits and plastic responses seem most likely to evolve in terrestrial embryos and larvae that must, or are able to, spend more extended periods on land; indeed, such mechanisms may be a key component of this ability. The benefits of urea excretion for early life stages should be balanced against the metabolic cost of urea synthesis (Shambaugh, 1977; Wright & Fyhn, 2001) in analyses of overall cost‐benefit trade‐offs across ecological and developmental transitions, including from terrestrial to aquatic environments in early development (Delia et al., 2019; Touchon & Warkentin, 2010; Warkentin, 1995) as well as from aquatic to terrestrial development at metamorphosis (Bouchard et al., 2016; Gomez‐Mestre et al., 2010; Touchon et al., 2013; Vonesh & Bolker, 2005).

Enzymatic mechanisms and genetic regulation of urea excretion have been studied in lungfishes under high risk of ammonia toxicity during terrestrial emersion (Chew et al., 2003, 2004; Loong et al., 2005, 2012) and in aquatic embryonic development of teleosts (Barimo et al., 2004; LeMoine & Walsh, 2013, 2015; Steele et al., 2001; Wright et al., 1995). However, few studies have explored such mechanisms in tetrapod lineages in the context of ammonia toxicity (Ip et al., 2012; Janssens, 1972; Wright & Wright, 1996) and the transition to terrestrial life (Brown et al., 1959; Weng et al., 2004). Understanding physiological mechanisms of plasticity may be important to understand evolutionary change (Ledón‐Rettig & Ragsdale, 2021; Suzuki & Nijhout, 2006). For labile traits that may change during individual lives (Rundle & Spicer, 2016), changes in physiological tolerance across environments in response to diverse abiotic factors (Braun et al., 2009; Hopkins et al., 2016; Mendez‐Sanchez & Burggren, 2017; Peña‐Villalobos et al., 2016) might also contribute to colonization of and survival in new environments (Kulkarni et al., 2017; Lande, 2015; Velotta & Cheviron, 2018). We suggest that repeated independent evolution of terrestrial development in frogs offers an excellent opportunity to study developmental mechanisms of physiological plasticity and their role in the reproductive colonization of land, considering ammonia toxicity as a common environmental threat and potential cue during early development.

## CONFLICT OF INTEREST

Authors acknowledge that there are no conflicts of interest.

## AUTHOR CONTRIBUTIONS

**Javier Méndez‐Narváez:** Conceptualization (lead); Formal analysis (lead); Funding acquisition (lead); Investigation (lead); Methodology (lead); Writing – original draft (lead); Writing – review & editing (equal). **Karen M. Warkentin:** Conceptualization (supporting); Formal analysis (supporting); Investigation (supporting); Writing – original draft (supporting); Writing – review & editing (equal).

## Data Availability

Data available from the Dryad Digital Repository: https://doi.org/10.5061/dryad.866t1g1r2.

## APPENDIX 1

**TABLE A1:** Concentration (mmol/L) of ammonia, urea‐N, and total N (ammonia +urea‐N) measured at standardized ages (days) under wet and dry conditions in developmental environments of four frog species: aquatic foam nests of *Engystomops pustulosus*, terrestrial foam nests of *Leptodactylus fragilis* and terrestrial jelly‐egg clutches of *Agalychnis callidryas* and *Hyalinobatrachium fleischmanni*

Age (days)	Below/above detection limit	Ammonia (mmol/L) Mean (SD), N		Urea‐N (mmol/L) Mean (SD), N		Total waste N (mmol/L) Mean (SD), N	
		Wet	Dry	Wet	Dry	Wet	Dry
*E. pustulosus*							
0	B A	0 (–), **11** 0.23 (–), **1**		– 0.67 (0.26), **12**		– 0.69 (0.30), **12**	
2.5	B A	– 1.68 (0.85), **14**	– 2.23 (0.98), **15**	0, **7** 0.42 (0.14), **7**	0, **5** 0.49 (0.12), **10**	– 1.90 (0.81), **14**	– 2.56 (1.06), **15**
4.5	B A	– 2.50 (1.05), **13**	– 3.40 (1.46), **15**	0, **12** 1.33 (–), **1**	0, **8** 0.50 (0.17), **7**	– 2.60 (1.13), **14**	– 3.63 (1.46), **15**
*L. fragilis*							
0	B A NA	0, **11** – –		– 7.16 (4.09), **10** NA, 1		– 7.16 (4.09), **10** NA, 1	
4.5	B A NA	– 2.16 (1.21), **18** –	– 1.75 (1.02), **22** NA, **1**	0, **4** 1.42 (0.60), **11** NA, **3**	0, **1** 2.81 (1.86), **16** NA, **5**	– 3.02 (1.16), **15** NA, **3**	– 4.02 (1.78), **17** NA, **5**
8.5	B A	– 4.35 (1.53), **9**	– 7.50 (4.78), **6**	0, **9** –	0, **6** –	– 4.35 (1.53)	– 7.50 (4.78), **6**
12.5	B A NA	– 6.76 (5.96), **18** –	– 53.45 (48.89), **14** –	0, **5** 9.37 (8.84), 8 NA, **5**	0, **3** 59.24 (71.04), **10** NA, **1**	– 13.24 (13.89), **13** NA, **5**	– 100.90 (97.35), **13** NA, **1**
*A. callidryas*							
0	B A	0, **9** –		– 0.70 (0.43), **9**		– 0.70 (0.43), **9**	
2.5	B A	0, **4** –	0, **7** 0.53 (–), **1**	0, **2** 0.27 (0.02), **2**	– 0.62 (0.25), **8**	– 0.13 (0.16), **4**	– 0.69 (0.20), **8**
4.5	B A NA	– 1.24 (0.38), **12** –	– 3.17 (0.75), **14** –	0, **12** –	0, **8** 0.98 (0.60), **5** NA, **1**	– 1.24 (0.38), **12** –	3.49 (1.02), **13** NA, **1**
5.5	B A	– 1.18 (0.46), **10**	– 4.27 (2.37), **11**	0, **9** 0.37 (‐), **1**	0, **10** 4.44 (NA), **1**	– 1.22 (0.47), **10**	– 4.68 (2.99), **11**
*H. fleischmanni*							
0	B A	0, **2** 0.40 (0.10), **4**		0, **6** –		0, **2** 0.40 (0.10), **4**	
5.5	B A	0, **5** –	– –	0, **4** 0.27 (–), **1**	– –	0, **4** 0.27 (–), **1**	
10.5	B A	– 1.23 (0.21), **13**	– 2.66 (0.87), **16**	0, **10** 0.36 (0.08), **3**	0, **5** 1.17 (0.97), **11**	– 1.32 (0.33), **13**	– 3.47 (1.61), **16**

Note

We assessed mean and standard deviation (SD) for concentrations above the detection limit (A); values below detection limit (B) are included only as the number of zeros. Where no samples were above or below detection limits, or SD could not be calculated, we indicate “–”. In some samples, ammonia was detected (A), but urea could not be quantified and is indicated as NA. Sample size (N) is indicated in bold.

**TABLE A2:** Experimental concentrations of total ammonia nitrogen (TAN, mmol/L) used for LC_50_ trials

Experimental concentrations								# Trials
1	2	3	4	5	6	7	8	
*E. pustulosus*								
2.3	3.7	4.6	38.2	109.2	–	–	–	*N* = 2
38.2	47.5	56.9	66.2	75.6	84.9	92.4	103.6	*N* = 10
*L. fragilis*								
37.7	61.9	85.5	109.2	132.9	156.5	180.2	550.9	*N* = 2
61.9	71.1	80.4	89.7	99.0	108.3	117.5	126.8	*N* = 3
89.7	95.0	100.3	105.6	110.9	116.2	121.5	126.8	*N* = 9
*A. callidryas*								
3.9	13.2	41.3	69.3	–	–	–	–	*N* = 1
3.9	10.6	17.3	23.9	30.7	37.3	44.0	50.7	*N* = 3
10.6	16.4	22.1	27.8	33.5	39.2	45.0	50.7	*N* = 3
22.1	26.2	30.3	34.4	38.5	42.6	46.7	50.8	*N* = 5
13.2	22.6	37.7	41.3	50.7	60.0	69.3	78.7	*N* = 1
*H. fleischmanni*								
0.9	4.9	9.0	13.1	17.2	21.3	25.4	29.5	*N* = 5

Note

Each trial used tadpoles from a single foam nest in *Engystomops pustulosus* and *Leptodactylus fragilis*, or pooled from several clutches in *Agalychnis callidryas* and *Hyalinobatrachium fleischmanni*, at the latest sampled age. We prepared solutions with NH_4_Cl covering the concentration range across species.

**TABLE A3:** Linear Mixed Effects Model (LMEM) in *Engystomops pustulosus* with significance levels after Likelihood Ratio Tests (LRT) of nested models, testing effects of age, treatment, and their interaction on concentration of ammonia and urea‐N

Model	LMEM model and *p*‐value	Permutated *p*‐value	Post hoc *p*‐value		
	**Ammonia**		**Age (days)**	**Wet**	**Dry**
Age	*χ* ^2^_2_ = 64,82, *p* < .0001	.0002	0–2.5	*t* = −5.86, *p* = .003	*t* = −5.86 *p* = .003
Treatment	*χ* ^2^_1_ = 6.93, *p* = .008	.01	0–4.5	*t* = −9.75, *p* = .003	*t* = −9.75, *p* = .0003
Interaction	*χ* ^2^_2_ = 0.49, *p* = .77	.76	2.5–4.5	*t* = −7.39, *p* = .0003	*t* = −7.39, *p* = .0003
			**Treatment**	**2.5 days**	**4.5 days**
			Wet–Dry	*t* = −2.62, *p* = .02	*t* = −2.62, *p* = .02
	**Urea**		**Age (days)**	**Wet**	**Dry**
Age	*χ* ^2^_2_ = 28.33, *p* < .0001	.0002	0–2.5	*t* = 4.06, *p* = .002	*t* = 4.06, *p* = .002
Treatment	*χ* ^2^_1_ = 4.76, *p* = .03	.03	0–4.5	*t* = 5.73, *p* = .001	*t* = 5.73, *p* = .001
Interaction	*χ* ^2^_2_ = 0.96, *p* = .61	.59	2.5–4.5	*t* = 2.42, *p* = .04	*t* = 2.42, *p* = .04
			**Treatment**	**2.5 days**	**4.5 days**
			Wet–Dry	*t* = −2.16, *p* = .05	*t* = −2.16, *p* = .05

Note

Ammonia and urea concentrations were rank‐transformed and permutated *p*‐values were obtained (5000 times) for fixed effects and for each pairwise comparison (adjusted for FDR), using the LMEM structure.

**TABLE A4:** Linear Models (LM) in *Leptodactylus fragilis* with significance levels, testing the effect of age, treatment, and their interaction on concentration of ammonia and urea‐N

Model	LM model and *p*‐value	Permutated *p*‐value	Post hoc *p*‐value			
	**Ammonia**		**Age (days)**	**Wet**	**Dry**	
Age Treatment Interaction	*F*_3,101_ = 143.3, *p* < .0001 *F*_1,101_ = 5.87, *p* = .03 *F*_3,101_ = 8.83, *p* < .0001	.0002 .01 .002	0–4.5 0–8.5 0–12.5 4.5–8.5 4.5–12.5 8.5–12.5	*t* = −6.86, *p *= .0003 *t* = −9.70, *p *= .0003 *t* = −11.94, *p *= .0003 *t* = −4.25, *p* = .0003 *t* = −5.77, *p *= .0003 *t* = −0.40, *p* = .73	*t* = −5.78, *p* = .0003 *t* = −10.07, *p* = .0003 *t* = −15.68, *p* = .0003 *t* = −6.46, *p* = .0003 *t* = −12.26, *p* = .0003 *t* = −2.66, *p *= .008	
			**Treatment**	**4.5 days**	**8.5 days**	**12.5 days**
			Wet–Dry	*t* = 1.55, *p* = .15	*t* = −1.42, *p* = .17	*t* = −5.29, *p *= .0003
	**Urea**		**Age (days)**	**Wet**	**Dry**	
Age Treatment Interaction	*F*_3,86_ = 36.7, *p* < .0001 *F*_1,86_ = 5.7, *p* = .019 *F*_3,86_ = 1.1, *p* = .35	.0002 .11 .35	0–4.5 0–8.5 0–12.5 4.5–8.5 4.5–12.5 8.5–12.5	*t* = 4.58, *p* = .0004 *t* = 7.27, *p* = .0004 *t* = 1.84, *p* = .08 *t* = 3.70, *p* = .0008 *t* = −3.21, *p* = .003 *t* = −6.35, *p* = .0004	*t* = 2.51, *p* = .02 *t* = 6.44, *p* = .0004 *t* = 0.40, *p* = .74 *t* = 5.08, *p* = .0004 *t* = −2.18, *p* = .05 *t* = −6.29, *p* = .0004	
			**Treatment**	**4.5 days**	**8.5 days**	**12.5 days**
			Wet–Dry	*t* = −2.60, *p* = .02	*t* = 0, *p* = 1	*t* = −1.48, *p* = .2

Note

Ammonia and urea concentrations were rank‐transformed and permutated *p*‐values were obtained (5000 times) for fixed effects and for each pairwise comparison (adjusted for FDR), using the LM structure.

**TABLE A5:** Linear Models (LM) in *Agalychnis callidryas* with significance levels, testing the effect of age, treatment, and their interaction on concentration of ammonia and urea‐N

Model	LM model and *p*‐value	Permutated *p*‐value	Post hoc *p*‐value			
	**Ammonia**		**Age (days)**	**Wet**	**Dry**	
Age Treatment Interaction	*F*_3,69_ = 334.59, *p* < .0001 *F*_1,69_ = 84.83, *p* < .0001 *F*_3,69_ = 14.52, *p* < .0001	.0002 .0002 .0002	0–2.5 0–4.5 0–5.5 2.5–4.5 2.5–5.5 4.5–5.5	*t* = 0, *p* = 1 *t* = −13.15, *p* = .0003 *t* = −12.25, *p* = .0003 *t* = −10.05, *p* = .0003 *t* = −9.51, *p* = .0003 *t* = 0.40, *p* = .76	*t* = −1.09, *p* = .35 *t* = −20.90, *p* = .0003 *t* = −20.31, *p* = .0003 *t* = −18.95, *p* = .0003 *t* = −18.50, *p* = .0003 *t* = −0.49, *p* = .73	
			**Treatment**	**2.5 days**	**4.5 days**	**5.5 days**
			Wet–Dry	*t* = −0.87, *p* = .49	*t* = −7.95, *p* = .0003	*t* = −8.02, *p* = .0003
	**Urea**		**Age (days)**	**Wet**	**Dry**	
Age Treatment Interaction	*F*_3,68_ = 32.47, *p* < .0001 *F*_1,68_ = 10.15, *p* = .002 *F*_3,68_ = 3.32, *p* = .02	.0002 .0006 .03	0–2.5 0–4.5 0–5.5 2.5–4.5 2.5–5.5 4.5–5.5	*t* = 3.14, *p* = .003 *t* = 6.89, *p* = .005 *t* = 6.08, *p* = .005 *t* = 1.98, *p* = .08 *t* = 1.52, *p* = .18 *t* = −0.57, *p* = .70	0–2.5 days, *t* = −0.01, *p* = 1 0–4.5 days, *t* = 4.02, *p* = .0005 0–5.5 days, *t* = 5.95, *p* = .0005 2.5–4.5 days, *t* = 3.89, *p* = .005 2.5–5.5 days, *t* = 5.77, *p* = .0005 4.5–5.5 days, *t* = 2.27, *p* = .04	
			**Treatment**	**2.5 days**	**4.5 days**	**5.5 days**
			Wet–Dry	*t* = −3.09, *p* = .003	*t* = −3.23, *p* = .002	*t* = −0.27, *p* = .90

Note

Ammonia and urea concentrations were rank‐transformed and permutated *p*‐values were obtained (5000 times) for fixed effects and for each pairwise comparison (adjusted for FDR), using the LM structure.

**TABLE A6:** Linear Model (LM) in *Hyalinobatrachium fleischmanni* with significance levels, testing differences in concentration of ammonia and urea‐N across ages and, after hatching competence, across treatments

Model	LM model and *p*‐value	Permutated *p*‐value	Post hoc *p*‐value		
	**Ammonia**		**Age**	**Wet**	**Dry**
Category	*F*_3,36_ = 122.93, *p* < .0001	.0002	0–5 days 0–10.5 days 5.5–10.5 days	*t* = 3.68, *p* = .002 *t* = 6.82, *p* = .0002 *t* = 10.63, *p* = .0002	– *t* = 13.30, *p* = .0002 t =16.78, *p* = .0002
			**Treatment**	**10.5 days**	
			Wet–Dry	*t* = −8.04, *p* = .0002	
	**Urea**		**Age**	**Wet**	**Dry**
Category	*F*_3,36_ = 6.59, *p* = 0.001	0.002	0–5 days 0–10.5 days 5.5–10.5 days	*t* = −0.65, *p* = .62 *t* = 0.98, *p* = .49 *t* = 0.17, *p* = .86	– *t* = 3.64, *p* = .008 *t* = 2.63, *p* = .02
			**Treatment** Wet–Dry	**10.5 days** *t* = −3.37, *p* = .01	

Note

Categories were shortly after oviposition and before and after hatching competence in the field under paternal care (wet: 0, 5.5, and 10.5 days), and after 5 days in the lab under dry conditions (10.5 days). Ammonia and urea‐N concentrations were rank‐transformed and permutated *p*‐values were obtained (5000 times) for fixed effects and for each pairwise comparison (adjusted for FDR), using the LM structure.

**TABLE A7:** Effect of wet and dry conditions (treatments) on the amount of ammonia, urea‐N, and total waste‐N (ammonia +urea‐N) present per individual, and urea‐N as a proportion of total waste‐N, measured at the latest sampling age in the foam nests of *Leptodactylus fragilis* and the perivitelline fluid of *Hyalinobatrachium fleischmanni*

Statistical model	*L. fragilis*	*H. fleischmanni*
Ammonia/individual ~ Treatment	*t*_26.4_ = −3.05, *p* = .005; *p* = .008	*t*_24.1_ = 1.06, *p* = .296; *p* = .325
Urea‐N/individual ~ Treatment	*W* = 43.5, *p* = .035; *p* = .049	*W* = 52, *p* = .016; *p* = .022
Total waste‐N/individual ~ Treatment	*t*_23.0_ = −3.30, *p* = .003; *p* = .005	*t*_26.3_ = −0.45, *p* = .657; *p* = .669
Urea‐N/total nitrogen ~ Treatment	*χ* ^2^ = 1.04, *p* = .30; *p* = .269	*χ* ^2^ = 5.59, *p* = .018; *p* = .006

Note

We transformed all data to account for zeros and obtained permutated *p*‐values using permutated *t*‐tests (5000 times). We also obtained parametric *p*‐values using *t*‐tests or Wilcoxon Rank Sum Tests (W) for amounts of excreted nitrogen wastes and a generalized linear mixed model (GLMM) with an underlying Beta error distribution and likelihood ratio test (LRT) for the proportion data.

**TABLE A8:** Effect of treatment (wet and dry conditions) and ammonia concentration (actual: Table A1; potential: ammonia +urea‐N) on the amount of ammonia and urea‐N present per individual in *Leptodactylus fragilis* and *Hyalinobatrachium fleischmanni*

Statistical models	*L. fragilis*
^1^Urea‐N/individual ~actual ammonia * treatment:	
Actual ammonia	*F*_1,22_ = 1.76, *p* = .198; *p* = .208
Treatment	*F*_1,22_ = 7.79, *p* = .011; *p* = .010
Interaction	*F*_1,22_ = 2.63, *p* = .119; *p* = .118
Urea‐N/individual ~potential ammonia * treatment:	
Potential ammonia	*F*_1,22_ = 5.67, *p* = .026; *p* = .015
Treatment	*F*_1,22_ = 4.39, *p* = .048; *p* = .049
Interaction	*F*_1,22_ = 4.76, *p* = .040; *p* = .037

Statistical models	*H. fleischmanni*
^2^ Urea‐N/individual ~actual ammonia * treatment:	
Actual ammonia	*F*_1,25_ = 3.09, *p* = .091; *p* = .114
Treatment	*F*_1,25_ = 5.09, *p* = .032; *p* = .035
Interaction	*F*_1,25_ = 3.23, *p* = .084; *p* = .085
Urea‐N/individual ~potential ammonia * Treatment	
Potential ammonia	*F*_1,25_ = 10.23, *p* = .004; *p* = .002
Treatment	*F*_1,25_ = 8.82, *p* = .006; *p* = .007
Interaction	*F*_1,25_ = 8.15, *p* = .008; *p* = .009

Note

We used an AIC approach to determine the LM’s that best explain the amount urea in developmental environments from two sets of models including only treatment, only ammonia (actual or potential) or both fixed factors with their interaction, followed by a permutation approach to obtain *p*‐values. When the best model included only treatment, we used results from Table A7. ^1, 2^ best models (AIC) had treatment as the only predictor, but *R* ^2^ was higher for full model with interaction: ^1^0.153 vs. 0.2673; ^2^ 0.209 vs. 0.299.

**TABLE A9:** Ammonia LC_50_ values or percent mortality at given concentrations (mmol/L) during acute (≤96 h) or chronic (>96 h) ammonia exposure in anurans (embryos and tadpoles) and fishes (embryo to adult stages)

Species	Exposure time	LC_50_ (mmol/L) or % mortality	Environmental ammonia	Developmental environment	Development stage	Reference
Anurans						
***Engystomops pustulosus***	96 h	53	2.5–3.4	Semi‐Terrestrial	Tadpole: early	This study
***Leptodactylus fragilis***	96 h	110	6.76–53.45	Terrestrial	Tadpole: early	This study
***Agalychnis callidryas***	96 h	36	1.18–4.27	Terrestrial	Tadpole: early	This study
***Hyalinobatrachium fleischmanni***	96 h	18	1.23–2.66	Terrestrial	Tadpole: early	This study
***Leptodactylus bufonius***	24 h	5 = 100%	4–40	Terrestrial	Tadpole	Shoemaker and McClanahan (1973)
***Gastrotheca riobambae***	24 h	0.1–0.025 = 0% 100–0.5 = 100%	5.2	Terrestrial	Tadpole	Alcocer et al. (1992)
*Xenopus laevis*	5 days	4.03* 3.11	0.14–1.49	Aquatic	Embryos	Schuytema and Nebeker (1999, 1999a), McDowell and McGregor (1979)
*Anaxyrus americanus*	96 h	0.97–2.81^a^		Aquatic	Tadpoles	Hecnar (1995)
*Bufo bufo*	96 h	6.43* =70%		Aquatic	Tadpoles	García‐Muñoz et al. (2011)
	96 h 7 days	27.45 26.37		Aquatic	Tadpoles	Xu and Oldham (1997)
*Bufo calamita*	96 h	6.43 = 80%		Aquatic	Tadpoles	García‐Muñoz et al. (2011)
		3.22 = 0%		Aquatic	Embryos	Ortiz‐Santaliestra and Marco (2015)
	7 days 12 days	3.22 = ~50% 3.22 = ~60%		Aquatic	Tadpoles	Ortiz‐Santaliestra and Marco (2015)
	15 days	8.06* =65.8%	0.004–0.26	Aquatic	Tadpoles	Miaud et al. (2011), Ortiz‐Santaliestra et al. (2006)
*Boana faber*	21 days	0.09 = 0 0.35 = 11%	0.12–0.30	Aquatic	Tadpoles	Ilha and Schiesari (2014)
*Discoglossus galganoi*	15 days	3.22* =31%	0.004–0.26	Aquatic	Tadpoles	Miaud et al. (2011), Ortiz‐Santaliestra et al. (2006)
*Euphlyctis cyanophlycti*	9 days	3.12 = >50%		Aquatic	Tadpoles	Bibi et al. (2016)
*Pseudacris regilla*	96 h	2.92* 4.29	0.14–1.49	Aquatic	Embryos	Schuytema and Nebeker (1999, 1999a), McDowell and McGregor (1979)
	10 days	1.77* 2.15	0.14–1.49	Aquatic	Tadpoles	
*Pseudacris triseriata*	96 h	1.21*		Aquatic	Tadpoles	Hecnar (1995)
*Pelobates cultripes*	15 days	3.22* =49%	0.05–3.6	Aquatic	Tadpoles	Miaud et al. (2011), Ortiz‐Santaliestra et al. (2006)
*Pelodytes ibericus*	96 h	6.43* =90%		Aquatic	Tadpoles	García‐Muñoz et al. (2011)
*Pelophylax perezi*	96 h	6.43* =0%		Aquatic	Tadpoles	García‐Muñoz et al. (2011)
*Lithobates pipiens*	96 h	1.60*		Aquatic	Tadpoles	Hecnar (1995)
*Lithobates clamitans*	96 h	2.30*		Aquatic	Tadpoles	Hecnar (1995)
*Lithobates silvatica*	7 days	0.62–1.24^b^ = ~50%	0.16–4.03	Aquatic	Tadpoles	Burgett et al. (2007)
*Rana aurora*	16 days	5.11		Aquatic	Tadpoles	Schuytema and Nebeker (1999, 1999b)
Fishes						
***Oncorhynchus mykiss***	96 h	0.18–11.50^c^			Juveniles	Thurston et al. (1981)
	38 days	1.19 = 2.5%			Sac Fry, 15 dph	Brinkman et al. (2009)
	50 days	1.85 = 40%			Embryos	de Solbé and Shurben (1989)
	90 days	1.19 = 77.5%			Fry, 52 dph	Brinkman et al. (2009)
***Opsanus beta***	96 h	63.6	0.008–0.01 (Nest)		Embryos	Barimo et al. (2004), Barimo and Walsh (2005)
		5.45			Larvae	Barimo and Walsh (2005)
		0.87			Juveniles	Barimo et al. (2004)
		9.7			Adults	Wang and Walsh (2000)
***Opsanus tau***	96 h	19.7			Adults	Wang and Walsh (2000)
***Opsanus notatus***	96 h	6			Adults	Wang and Walsh (2000)
***Pelteobagrus fulvidraco***	96 h	1.77–4.90^e^			Adults	Zhang et al. (2012)
***Monopterus albus***	96 h	193.2			Adults	Ip, Tay, et al. (2004)
*Clarias gariepinus*	5 days 96 h	100 = 0% 380			Adults	Ip, Zubaidah, et al. (2004)
***Protopterus dolloi***	6 d	100 = 0%			Adults	Chew et al. (2004)
***Periophthalmodon schlosseri***	Weeks 96 h	100 = 0% 120			Adults	Peng et al. (1998), Ip, Leong, et al. (2005), Ip, Peh, et al. (2005)
*Heteropneustes fossilis*	Weeks 96 h	75 = 0% 100 = 100%			Adults	Saha and Ratha (1994); Chew et al. (2020)
***Alcolapia grahami***	24 h	0.77 0.75			Juveniles Adults	Walsh et al. (1993)
*Pimephales promelas*	96 h	2.42–7.70^d^ 0.42–18.15^c^			Adults	Thurston et al. (1983), Thurston et al. (1981)
*Notropis topeka*	96 h	1.15 2.43			Juveniles Adults	Adelman et al. (2009)
*Notropis* spp.	96 h	1.19–3.41^f^			Adults	Adelman et al. (2009)

Note

Names in bold indicates species where urea excretion has been reported. All ammonia concentrations are presented based on total ammonia nitrogen, mostly from NH_4_Cl; asterisks indicate values from NH_4_NO_3_. Ammonia level or ranges measured in developmental environments are included where available.

^a^ For different populations

^b^ For different life stages

^c^ For different pH solutions

^d^ For different temperatures

^e^ For different adult sizes

^f^ For different species, cited in Adelman et al. (2009).
