# Supplementary material for: Reproductive colonization of land by frogs: Embryos and larvae excrete urea to avoid ammonia toxicity
Source: Ecol Evol. 2022 Feb 14;12(2):e8570. doi: 10.1002/ece3.8570 (PMC8843769; doi:10.1002/ece3.8570)
Supplement: Supplementary file 1 [file ECE3-12-e8570-s001.docx]

**Appendix**

**Table A1**. Concentration (mmol/L) of ammonia, urea-N, and total N (ammonia + urea-N) measured at standardized ages (days) under wet and dry conditions in developmental environments of four frog species: aquatic foam nests of *Engystomops pustulosus*, terrestrial foam nests of *Leptodactylus fragilis* and terrestrial jelly-egg clutches of *Agalychnis callidryas* and *Hyalinobatrachium fleischmanni.* We assessed mean and standard deviation (SD) for concentrations above the detection limit (A); values below detection limit (B) are included only as the number of zeros. Where no samples were above or below detection limits, or SD could not be calculated, we indicate “–”. In some samples, ammonia was detected (A), but urea could not be quantified and is indicated as NA. Sample size (**N**) is indicated in bold.

| Age (days) | Below/Above  Detection  Limit | Ammonia (mmol/L):  mean (SD), **N** | | Urea-N (mmol/L)  mean (SD), **N** | | Total waste N (mmol/L)  mean (SD), **N** | |
| --- | --- | --- | --- | --- | --- | --- | --- |
|  |  | Wet | Dry | Wet | Dry | Wet | Dry |
|  |  | *E. pustulosus* | | | | | |
| 0 | B  A | 0 (–), **11**  0.23 (–), **1** | | –  0.67 (0.26), **12** | | –  0.69 (0.30), **12** | |
| 2.5 | B  A | –  1.68 (0.85), **14** | –  2.23 (0.98), **15** | 0, **7**  0.42 (0.14), **7** | 0, **5**  0.49 (0.12), **10** | –  1.90 (0.81), **14** | –  2.56 (1.06), **15** |
| 4.5 | B  A | –  2.50 (1.05), **13** | –  3.40 (1.46), **15** | 0, **12**  1.33 (–), **1** | 0, **8**  0.50 (0.17), **7** | –  2.60 (1.13), **14** | –  3.63 (1.46), **15** |
|  |  | *L. fragilis* | | | | | |
| 0 | B  A  NA | 0, **11**  –  – | | –  7.16 (4.09), **10**  NA, 1 | | –  7.16 (4.09), **10**  NA, 1 | |
| 4.5 | B  A  NA | –  2.16 (1.21), **18**  – | –  1.75 (1.02), **22**  NA, **1** | 0, **4**  1.42 (0.60), **11**  NA, **3** | 0, **1**  2.81 (1.86), **16**  NA, **5** | –  3.02 (1.16), **15**  NA, **3** | –  4.02 (1.78), **17**  NA, **5** |
| 8.5 | B  A | –  4.35 (1.53), **9** | –  7.50 (4.78), **6** | 0, **9**  – | 0, **6**  – | –  4.35 (1.53) | –  7.50 (4.78), **6** |
| 12.5 | B  A  NA | –  6.76 (5.96), **18**  – | –  53.45 (48.89), **14**  – | 0, **5**  9.37 (8.84), 8  NA, **5** | 0, **3**  59.24 (71.04), **10**  NA, **1** | –  13.24 (13.89), **13**  NA, **5** | –  100.90 (97.35), **13**  NA, **1** |
|  |  | *A. callidryas* | | | | | |
| 0 | B  A | 0, **9**  – | | –  0.70 (0.43), **9** | | –  0.70 (0.43), **9** | |
| 2.5 | B  A | 0, **4**  – | 0, **7**  0.53 (–), **1** | 0, **2**  0.27 (0.02), **2** | –  0.62 (0.25), **8** | –  0.13 (0.16), **4** | –  0.69 (0.20), **8** |
| 4.5 | B  A  NA | –  1.24 (0.38), **12**  – | –  3.17 (0.75), **14**  – | 0, **12**  – | 0, **8**  0.98 (0.60), **5**  NA, **1** | –  1.24 (0.38), **12**  – | 3.49 (1.02), **13**  NA, **1** |
| 5.5 | B  A | –  1.18 (0.46), **10** | –  4.27 (2.37), **11** | 0, **9**  0.37 (-), **1** | 0, **10**  4.44 (NA), **1** | –  1.22 (0.47), **10** | –  4.68 (2.99), **11** |
|  |  | *H. fleischmanni* | | | | | |
| 0 | B  A | 0, **2**  0.40 (0.10), **4** | | 0, **6**  – | | 0, **2**  0.40 (0.10), **4** | |
| 5.5 | B  A | 0, **5**  – | –  – | 0, **4**  0.27 (–), **1** | –  – | 0, **4**  0.27 (–), **1** |  |
| 10.5 | B  A | –  1.23 (0.21), **13** | –  2.66 (0.87), **16** | 0, **10**  0.36 (0.08), **3** | 0, **5**  1.17 (0.97), **11** | –  1.32 (0.33), **13** | –  3.47 (1.61), **16** |

**Table A2.** Experimental concentrations of total ammonia nitrogen (TAN, mmol/L) used for LC_50_ trials. Each trial used tadpoles from a single foam nest in *Engystomops pustulosus* and *Leptodactylus fragilis*, or pooled from several clutches in *Agalychnis callidryas* and *Hyalinobatrachium fleischmanni*, at the latest sampled age*.* We prepared solutions with NH_4_Cl covering the concentration range across species.

| Experimental concentrations | | | | | | | | | # Trials |
| --- | --- | --- | --- | --- | --- | --- | --- | --- | --- |
| 1 | | 2 | 3 | 4 | 5 | 6 | 7 | 8 |  |
| *E. pustulosus* | | | | | | | | | |
| 2.3 | 3.7 | | 4.6 | 38.2 | 109.2 | – | – | – | N=2 |
| 38.2 | 47.5 | | 56.9 | 66.2 | 75.6 | 84.9 | 92.4 | 103.6 | N=10 |
| *L. fragilis* | | | | | | | | | |
| 37.7 | 61.9 | | 85.5 | 109.2 | 132.9 | 156.5 | 180.2 | 550.9 | N=2 |
| 61.9 | 71.1 | | 80.4 | 89.7 | 99.0 | 108.3 | 117.5 | 126.8 | N=3 |
| 89.7 | 95.0 | | 100.3 | 105.6 | 110.9 | 116.2 | 121.5 | 126.8 | N=9 |
| *A. callidryas* | | | | | | | | | |
| 3.9 | 13.2 | | 41.3 | 69.3 | – | – | – | – | N=1 |
| 3.9 | 10.6 | | 17.3 | 23.9 | 30.7 | 37.3 | 44.0 | 50.7 | N=3 |
| 10.6 | 16.4 | | 22.1 | 27.8 | 33.5 | 39.2 | 45.0 | 50.7 | N=3 |
| 22.1 | 26.2 | | 30.3 | 34.4 | 38.5 | 42.6 | 46.7 | 50.8 | N=5 |
| 13.2 | 22.6 | | 37.7 | 41.3 | 50.7 | 60.0 | 69.3 | 78.7 | N=1 |
| *H. fleischmanni* | | | | | | | | | |
| 0.9 | 4.9 | | 9.0 | 13.1 | 17.2 | 21.3 | 25.4 | 29.5 | N=5 |

**Table A3.** Linear Mixed Effects Model (LMEM) in *Engystomops pustulosus* with significance levels after Likelihood Ratio Tests (LRT) of nested models, testing effects of age, treatment, and their interaction on concentration of ammonia and urea-N. Ammonia and urea concentrations were rank-transformed and permutated *p*-values were obtained (5000 times) for fixed effects and for each pairwise comparison (adjusted for FDR), using the LMEM structure.

| Model | LMEM model and  p-value | Permutated *p*-value | Post hoc *p*-value | | |
| --- | --- | --- | --- | --- | --- |
| Age  Treatment  Interaction | Ammonia  X^2^_2_ = 64,82, p < 0.0001  X^2^_1_ = 6.93, p = 0.008  X^2^_2_ = 0.49, p = 0.77 | 0.0002  0.01  0.76 | **Age (days)**  0–2.5  0–4.5  2.5–4.5 | **Wet**  t = -5.86, *p* = 0.003  t = -9.75, *p* = 0.003  t = -7.39, *p* = 0.0003 | **Dry**  t = -5.86 *p* = 0.003  t = -9.75, *p* = 0.0003  t = -7.39, *p* = 0.0003 |
|  |  |  | **Treatment**  Wet–Dry | **2.5 days**  t = -2.62, *p* = 0.02 | **4.5 days**  t = -2.62, *p* = 0.02 |
| Age  Treatment  Interaction | Urea  X^2^_2_ = 28.33, p < 0.0001  X^2^_1_ = 4.76, p = 0.03  X^2^_2_ = 0.96, p = 0.61 | 0.0002  0.03  0.59 | **Age (days)**  0–2.5  0–4.5  2.5–4.5 | **Wet**  t = 4.06, *p* = 0.002  t = 5.73, *p* = 0.001  t = 2.42, *p* = 0.04 | **Dry**  t = 4.06, *p* = 0.002  t = 5.73, *p* = 0.001  t = 2.42, *p* = 0.04 |
|  |  |  | **Treatment**  Wet–Dry | **2.5 days**  t = -2.16, *p* = 0.05 | **4.5 days**  t = -2.16, *p* = 0.05 |

**Table A4**. Linear Models (LM) in *Leptodactylus fragilis* with significance levels, testing the effect of age, treatment, and their interaction on concentration of ammonia and urea-N. Ammonia and urea concentrations were rank-transformed and permutated *p*-values were obtained (5000 times) for fixed effects and for each pairwise comparison (adjusted for FDR), using the LM structure.

| Model | LM model and  p-value | Permutated *p*-value | Post hoc *p*-value | | | |
| --- | --- | --- | --- | --- | --- | --- |
| Age  Treatment  Interaction | **Ammonia**  F_3,101_ = 143.3, p < 0.0001  F_1,101_ = 5.87, p = 0.03  F_3,101_ = 8.83, p < 0.0001 | 0.0002  0.01  0.002 | **Age (days)**  0–4.5  0–8.5  0–12.5  4.5–8.5  4.5–12.5  8.5–12.5 | **Wet**  t = -6.86, *p =* 0.0003  t = -9.70, *p =* 0.0003  t = -11.94, *p =* 0.0003  t = -4.25, *p* = 0.0003  t = -5.77, *p =* 0.0003  t = -0.40, *p* = 0.73 | **Dry**  t = -5.78, *p* = 0.0003  t = -10.07, *p* = 0.0003  t = -15.68, *p* = 0.0003  t = -6.46, *p* = 0.0003  t = -12.26, *p* = 0.0003  t = -2.66, *p* *=* 0.008 | |
|  |  |  | **Treatment**  Wet–Dry | **4.5 days**  t = 1.55, *p* = 0.15 | **8.5 days**  t = -1.42, *p* = 0.17 | **12.5 days**  t = -5.29, *p =* 0.0003 |
| Age  Treatment  Interaction | **Urea**  F_3,86_ = 36.7, p < 0.0001  F_1,86_ = 5.7, p = 0.019  F_3,86_ = 1.1, p = 0.35 | 0.0002  0.11  0.35 | **Age (days)**  0–4.5  0–8.5  0–12.5  4.5–8.5  4.5–12.5  8.5–12.5 | **Wet**  t = 4.58, *p* = 0.0004  t = 7.27, *p* = 0.0004  t = 1.84, *p* = 0.08  t = 3.70, *p* = 0.0008  t = -3.21, *p* = 0.003  t = -6.35, *p* = 0.0004 | **Dry**  t = 2.51, *p* = 0.02  t = 6.44, *p* = 0.0004  t = 0.40, *p* = 0.74  t = 5.08, *p* = 0.0004  t = -2.18, *p* = 0.05  t = -6.29, *p* = 0.0004 | |
|  |  |  | **Treatment**  Wet–Dry | **4.5 days**  t = -2.60, *p* = 0.02 | **8.5 days**  t = 0, *p* = 1 | **12.5 days**  t = -1.48, *p* = 0.2 |

**Table A5**. Linear Models (LM) in *Agalychnis callidryas* with significance levels, testing the effect of age, treatment, and their interaction on concentration of ammonia and urea-N. Ammonia and urea concentrations were rank-transformed and permutated *p*-values were obtained (5000 times) for fixed effects and for each pairwise comparison (adjusted for FDR), using the LM structure.

| Model | LM model and  p-value | Permutated *p*-value | Post hoc *p*-value | | | |
| --- | --- | --- | --- | --- | --- | --- |
| Age  Treatment  Interaction | **Ammonia**  F_3,69_ = 334.59, p < 0.0001  F_1,69_ = 84.83, p < 0.0001  F_3,69_ = 14.52, p < 0.0001 | 0.0002  0.0002  0.0002 | **Age (days)**  0–2.5  0–4.5  0–5.5  2.5–4.5  2.5–5.5  4.5–5.5 | **Wet**  t = 0, *p* = 1  t = -13.15, *p* = 0.0003  t = -12.25, *p* = 0.0003  t = -10.05, *p* = 0.0003  t = -9.51, *p* = 0.0003  t = 0.40, *p* = 0.76 | **Dry**  t = -1.09, *p* = 0.35  t = -20.90, *p* = 0.0003  t = -20.31, *p* = 0.0003  t = -18.95, *p* = 0.0003  t = -18.50, *p* = 0.0003  t = -0.49, *p* = 0.73 | |
|  |  |  | **Treatment** | **2.5 days** | **4.5 days** | **5.5 days** |
|  |  |  | Wet–Dry | t = -0.87, *p* = 0.49 | t = -7.95, *p* = 0.0003 | t = -8.02, *p* = 0.0003 |
| Age  Treatment  Interaction | **Urea**  F_3,68_ = 32.47, p < 0.0001  F_1,68_ = 10.15, p = 0.002  F_3,68_ = 3.32, p = 0.02 | 0.0002  0.0006  0.03 | **Age (days)**  0–2.5  0–4.5  0–5.5  2.5–4.5  2.5–5.5  4.5–5.5 | **Wet**  t = 3.14, *p* = 0.003  t = 6.89, *p* = 0.005  t = 6.08, *p* = 0.005  t = 1.98, *p* = 0.08  t = 1.52, *p* = 0.18  t = -0.57, *p* =0.70 | **Dry**  0–2.5 d, t = -0.01, *p* = 1  0–4.5 d, t = 4.02, *p* = 0.0005  0–5.5 d, t = 5.95, *p* = 0.0005  2.5–4.5 d, t = 3.89, *p* = 0.005  2.5–5.5 d, t = 5.77, *p* = 0.0005  4.5–5.5 d, t = 2.27, *p* = 0.04 | |
|  |  |  | **Treatment** | **2.5 days** | **4.5 days** | **5.5 days** |
|  |  |  | Wet–Dry | t = -3.09, *p* = 0.003 | t = -3.23, *p* = 0.002 | t = -0.27, *p* = 0.90 |

**Table A6.** Linear Model (LM) in *Hyalinobatrachium fleischmanni* with significance levels, testing differences in concentration of ammonia and urea-N across ages and, after hatching competence, across treatments. Categories were shortly after oviposition and before and after hatching competence in the field under paternal care (wet: 0, 5.5, and 10.5 d), and after 5 days in the lab under dry conditions (10.5 d). Ammonia and urea-N concentrations were rank-transformed and permutated *p*-values were obtained (5000 times) for fixed effects and for each pairwise comparison (adjusted for FDR), using the LM structure.

| Model | LM model and  p-value | Permutated *p*‑value | Post hoc *p*-value | | |
| --- | --- | --- | --- | --- | --- |
| Category | **Ammonia**  F_3,36_ = 122.93, p < 0.0001 | 0.0002 | **Age**  0–5 d  0–10.5 d  5.5 –10.5 d | **Wet**  t = 3.68, *p* = 0.002  t = 6.82, *p* = 0.0002  t = 10.63, *p* = 0.0002 | **Dry**  --  t = 13.30, *p* = 0.0002  t =16.78, *p* = 0.0002 |
|  |  |  | **Treatment**  Wet–Dry | **10.5 days**  t = -8.04, *p* = 0.0002 | |
| Category | **Urea**  F_3,36_ = 6.59, p = 0.001 | 0.002 | **Age**  0–5 d  0–10.5 d  5.5 –10.5 d | **Wet**  t = -0.65, *p* = 0.62  t = 0.98, *p* = 0.49  t = 0.17, *p* = 0.86 | **Dry**  --  t = 3.64, *p* = 0.008  t = 2.63, *p* = 0.02 |
|  |  |  | **Treatment**  Wet–Dry | **10.5 days**  t = -3.37, *p* = 0.01 | |

**Table A7.** Effect of wet and dry conditions (treatments) on the amount of ammonia, urea-N, and total waste-N (ammonia + urea-N) present per individual, and urea-N as a proportion of total waste-N, measured at the latest sampling age in the foam nests of *Leptodactylus fragilis* and the perivitelline fluid of *Hyalinobatrachium fleischmanni*. We transformed all data to account for zeros and obtained permutated *p*-values using permutated t-tests (5000 times). We also obtained parametric p-values using t-tests or Wilcoxon Rank Sum Tests (W) for amounts of excreted nitrogen wastes and a generalized linear mixed model (GLMM) with an underlying Beta error distribution and likelihood ratio test (LRT) for the proportion data.

| Statistical model | *L. fragilis* | *H. fleischmanni* |
| --- | --- | --- |
| Ammonia/individual ~ Treatment | t_26.4_ = -3.05, p = 0.005; *p* = 0.008 | t _24.1_ = 1.06, p = 0.296; *p* = 0.325 |
| Urea-N/individual ~ Treatment | W = 43.5, p = 0.035; *p* = 0.049 | W = 52, p = 0.016; *p* = 0.022 |
| Total waste-N/individual ~ Treatment | t_23.0_ = -3.30, p = 0.003; *p* = 0.005 | t_26.3_ = -0.45, p = 0.657; *p* = 0.669 |
| Urea-N/total nitrogen ~ Treatment | χ2 = 1.04, p = 0.30; *p* = 0.269 | χ2 = 5.59, p = 0.018; *p* = 0.006 |

**Table A8.** Effect of treatment (wet and dry conditions) and ammonia concentration (actual: Table A1; potential: ammonia + urea-N) on the amount of ammonia and urea-N present per individual in *Leptodactylus fragilis* and *Hyalinobatrachium fleischmanni*. We used an AIC approach to determine the LM’s that best explain the amount urea in developmental environments from two sets of models including only treatment, only ammonia (actual or potential) or both fixed factors with their interaction, followed by a permutation approach to obtain *p*-values. When the best model included only treatment, we used results from Table A7.

| Statistical models | *L. fragilis* |
| --- | --- |
| **^1^ Urea-N/individual ~ actual ammonia * treatment:**  actual ammonia  treatment  interaction  **Urea-N/individual ~ potential ammonia * treatment:**  potential ammonia  treatment  interaction | F_1,22_ = 1.76, p = 0.198; *p* = 0.208  F_1,22_ = 7.79, p = 0.011; *p* = 0.010  F_1,22_ = 2.63, p = 0.119; *p* = 0.118  F_1,22_ = 5.67, p = 0.026; *p* = 0.015  F_1,22_ = 4.39, p = 0.048; *p* = 0.049  F_1,22_ = 4.76, p = 0.040; *p* = 0.037 |
| Statistical models | *H. fleischmanni* |
| **^2^ Urea-N/individual ~ actual ammonia * treatment:**  actual ammonia  treatment  interaction  **Urea-N/individual ~ potential ammonia * Treatment**  potential ammonia  treatment  interaction | F_1,25_ = 3.09, p = 0.091; *p* = 0.114  F_1,25_ = 5.09, p = 0.032; *p* = 0.035  F_1,25_ = 3.23, p = 0.084; *p* = 0.085  F_1,25_ = 10.23, p = 0.004; *p* = 0.002  F_1,25_ = 8.82, p = 0.006; *p* = 0.007  F_1,25_ = 8.15, p = 0.008; *p* = 0.009 |

^1, 2^ best models (AIC) had treatment as the only predictor, but R^2^ was higher for full model with interaction: ^1^0.153 vs 0.2673; ^2^ 0.209 vs 0.299

**Table A9.** Ammonia LC_50_ values or percent mortality at given concentrations (mmol/L) during acute (≤ 96 h) or chronic (> 96 h) ammonia exposure in anurans (embryos and tadpoles) and fishes (embryo to adult stages). Names in bold indicates species where urea excretion has been reported. All ammonia concentrations are presented based on total ammonia nitrogen, mostly from NH_4_Cl; asterisks indicate values from NH_4_NO_3_. Ammonia level or ranges measured in developmental environments are included where available.

| Species | Exposure time | LC_50_ (mmol/L)  or % mortality | Environmental ammonia | Developmental environment | Development stage | Reference |
| --- | --- | --- | --- | --- | --- | --- |
| Anurans | | | | | | |
| ***Engystomops pustulosus*** | 96 h | 53 | 2.5–3.4 | Semi-Terrestrial | Tadpole: early | This study |
| ***Leptodactylus fragilis*** | 96 h | 110 | 6.76–53.45 | Terrestrial | Tadpole: early | This study |
| ***Agalychnis callidryas*** | 96 h | 36 | 1.18–4.27 | Terrestrial | Tadpole: early | This study |
| ***Hyalinobatrachium fleischmanni*** | 96 h | 18 | 1.23–2.66 | Terrestrial | Tadpole: early | This study |
| ***Leptodactylus bufonius*** | 24 h | 5 = 100% | 4–40 | Terrestrial | Tadpole | Shoemaker & McClanahan, 1973 |
| ***Gastrotheca riobambae*** | 24 h | 0.1–0.025 = 0%  100–0.5 = 100% | 5.2 | Terrestrial | Tadpole | Alcocer et al., 1992 |
| *Xenopus laevis* | 5 d | 4.03*  3.11 | 0.14–1.49 | Aquatic | Embryos | Schuytema & Nebeker, 1999a; McDowell & McGregor, 1979 |
| *Anaxyrus americanus* | 96 h | 0.97–2.81^1^ |  | Aquatic | Tadpoles | Hecnar, 1995 |
| *Bufo bufo* | 96 h | 6.43* = 70% |  | Aquatic | Tadpoles | García-Muñoz et al., 2011 |
|  | 96 h  7 d | 27.45  26.37 |  | Aquatic | Tadpoles | Xu & Oldham, 1997 |
| *Bufo calamita* | 96 h | 6.43 = 80% |  | Aquatic | Tadpoles | García-Muñoz et al., 2011 |
|  |  | 3.22 = 0% |  | Aquatic | Embryos | Ortiz-Santaliestra & Marco, 2015 |
|  | 7 d  12 d | 3.22 = ~50%  3.22 = ~60% |  | Aquatic | Tadpoles | Ortiz-Santaliestra & Marco, 2015 |
|  | 15 d | 8.06* = 65.8% | 0.004–0.26 | Aquatic | Tadpoles | Miaud et al., 2011; Ortiz-Santaliestra et al., 2006 |
| *Boana faber* | 21 d | 0.09 = 0  0.35 = 11% | 0.12–0.30 | Aquatic | Tadpoles | Ilha & Schiesari, 2014 |
| *Discoglossus galganoi* | 15 d | 3.22* = 31% | 0.004–0.26 | Aquatic | Tadpoles | Miaud et al., 2011; Ortiz-Santaliestra et al., 2006 |
| *Euphlyctis cyanophlycti* | 9 d | 3.12 = >50% |  | Aquatic | Tadpoles | Bibi et al., 2016 |
| *Pseudacris regilla* | 96 h | 2.92*  4.29 | 0.14–1.49 | Aquatic | Embryos | Schuytema & Nebeker, 1999a; McDowell & McGregor, 1979 |
|  | 10 d | 1.77*  2.15 | 0.14–1.49 | Aquatic | Tadpoles |  |
| *Pseudacris triseriata* | 96 h | 1.21* |  | Aquatic | Tadpoles | Hecnar, 1995 |
| *Pelobates cultripes* | 15 d | 3.22* = 49% | 0.05–3.6 | Aquatic | Tadpoles | Miaud et al., 2011; Ortiz-Santaliestra et al., 2006 |
| *Pelodytes ibericus* | 96 h | 6.43* = 90% |  | Aquatic | Tadpoles | García-Muñoz et al., 2011 |
| *Pelophylax perezi* | 96 h | 6.43* = 0% |  | Aquatic | Tadpoles | García-Muñoz et al., 2011 |
| *Lithobates pipiens* | 96 h | 1.60* |  | Aquatic | Tadpoles | Hecnar, 1995 |
| *Lithobates clamitans* | 96 h | 2.30* |  | Aquatic | Tadpoles | Hecnar, 1995 |
| *Lithobates silvatica* | 7 d | 0.62–1.24^2^ = ~50% | 0.16–4.03 | Aquatic | Tadpoles | Burgett et al., 2007 |
| *Rana aurora* | 16 d | 5.11 |  | Aquatic | Tadpoles | Schuytema & Nebeker, 1999b |
| Fishes | | | | | | |
| ***Oncorhynchus mykiss*** | 96 h | 0.18–11.50^3^ |  |  | Juveniles | Thurston et al., 1981 |
|  | 38 d | 1.19 = 2.5% |  |  | Sac Fry, 15 dph | Brinkman et al., 2009 |
|  | 50 d | 1.85 = 40% |  |  | Embryos | Solbé & Shurben, 1989 |
|  | 90 d | 1.19 = 77.5% |  |  | Fry, 52 dph | Brinkman et al., 2009 |
| ***Opsanus beta*** | 96 h | 63.6 | 0.008–­0.01 (Nest) |  | Embryos | Barimo et al., 2004; Barimo & Walsh, 2005 |
|  |  | 5.45 |  |  | Larvae | Barimo and Walsh 2005 |
|  |  | 0.87 |  |  | Juveniles | Barimo et al. 2004 |
|  |  | 9.7 |  |  | Adults | Wang & Walsh, 2000 |
| ***Opsanus tau*** | 96 h | 19.7 |  |  | Adults | Wang and Walsh, 2000 |
| ***Opsanus notatus*** | 96 h | 6 |  |  | Adults | Wang and Walsh, 2000 |
| ***Pelteobagrus fulvidraco*** | 96 h | 1.77–4.90^5^ |  |  | Adults | Zhang et al., 2012 |
| ***Monopterus albus*** | 96 h | 193.2 |  |  | Adults | Ip, Tay, et al., 2004 |
| *Clarias gariepinus* | 5 d  96 h | 100 = 0%  380 |  |  | Adults | Ip, Zubaidah, et al., 2004 |
| ***Protopterus dolloi*** | 6 d | 100 = 0% |  |  | Adults | Chew et al., 2004 |
| ***Periophthalmodon schlosseri*** | Weeks  96 h | 100 = 0%  120 |  |  | Adults | Peng et al., 1998;  Ip et al., 2005 |
| *Heteropneustes fossilis* | Weeks  96 h | 75 = 0%  100 = 100% |  |  | Adults | Saha & Ratha, 1994;  Chew et al., 2020 |
| ***Alcolapia grahami*** | 24 h | 0.77  0.75 |  |  | Juveniles  Adults | Walsh et al., 1993 |
| *Pimephales promelas* | 96 h | 2.42–7.70^4^  0.42–18.15^3^ |  |  | Adults | Thurston et al., 1983; Thurston et al., 1981 |
| *Notropis topeka* | 96 h | 1.15  2.43 |  |  | Juveniles  Adults | Adelman et al., 2009 |
| *Notropis spp.* | 96 h | 1.19–3.41^6^ |  |  | Adults | Adelman et al. 2009 |

^1^ For different populations

^2^ For different life stages

^3^ For different pH solutions

^4^ For different temperatures

^5^ For different adult sizes

^6^ For different species, cited in Adelman et al. 2009.
